# The Intrinsic Connectome of the Rat Amygdala

**DOI:** 10.3389/fncir.2012.00081

**Published:** 2012-12-11

**Authors:** Oliver Schmitt, Peter Eipert, Konstanze Philipp, Richard Kettlitz, Georg Fuellen, Andreas Wree

**Affiliations:** ^1^Department of Anatomy, University of RostockRostock, Germany; ^2^Institute for Biostatistics and Informatics in Medicine and Ageing Research, University of RostockRostock, Germany

**Keywords:** amygdala, connectome, tract-tracing, network analysis, stereotaxic atlas, visualization, rat brain, simulation

## Abstract

The connectomes of nervous systems or parts there of are becoming important subjects of study as the amount of connectivity data increases. Because most tract-tracing studies are performed on the rat, we conducted a comprehensive analysis of the amygdala connectome of this species resulting in a meta-study. The data were imported into the *neuroVIISAS* system, where regions of the connectome are organized in a controlled ontology and network analysis can be performed. A weighted digraph represents the bilateral intrinsic (connections of regions of the amygdala) and extrinsic (connections of regions of the amygdala to non-amygdaloid regions) connectome of the amygdala. Its structure as well as its local and global network parameters depend on the arrangement of neuronal entities in the ontology. The intrinsic amygdala connectome is a small-world and scale-free network. The anterior cortical nucleus (72 in- and out-going edges), the posterior nucleus (45), and the anterior basomedial nucleus (44) are the nuclear regions that posses most in- and outdegrees. The posterior nucleus turns out to be the most important nucleus of the intrinsic amygdala network since its Shapley rate is minimal. Within the intrinsic amygdala, regions were determined that are essential for network integrity. These regions are important for behavioral (processing of emotions and motivation) and functional (memory) performances of the amygdala as reported in other studies.

## 1 Introduction

A *connectome* is a level-dependent representation of connections between biological entities. Levels could be molecular, cellular (micro level), cohesive structural and/or functional ensembles of cells (meso level), or distinct groups of cohesive ensembles (macro level). With regard to the nervous system of the rat, we investigated data of a partial connectome of the central nervous system, the connectome of the amygdala (amygdaloid complex), and its extrinsic efferent targets as well as extrinsic afferent sources at the meso level of nuclei and their subdivisions. The intrinsic and extrinsic amygdala data are part of an evaluation of about 2100 publications conducted for this study and contain exclusively tract-tracing data of the rat nervous system (peripheral nervous system, spinal cord, brain). One rationale of this study is to uncover the connectivity of the intrinsic amygdala network of the rat based on high-resolution tract-tracing data. So far, the connectome of the intrinsic amygdala network has not been investigated in terms of network measures, graph theoretical quantities, and multivariate statistics. After making these connectome data accessible through a weighted connectivity matrix, specific questions concerning motifs, reciprocity, and most strongly connected regions upon others can be addressed.

Connectivity data sets of parts of nervous systems have been developed and analyzed by several groups (Felleman and Essen, 1991; Young, 1992, 1993; Scannell et al., 1999; Sporns et al., 2000, 2002; Sporns and Kötter, 2004; Sporns and Zwi, 2004; Honey et al., 2007; Modha and Singh, 2010; Sugar et al., 2011). Most of these connections are compiled into by meta-studies of tract-tracing publications. The sources of information in this study originate exclusively from peer-reviewed tract-tracing publications where anterograde and/or retrograde tracers were applied (lesion studies were not evaluated). Only those publications were considered that describe connectivity in adult rats. In our case, connections between neuronal regions are handled with the help of a consistent neuroontology (Schmitt and Eipert, 2012) that can be updated frequently in parallel to the fast progress of the identification of connections in tract-tracing publications.

Partial connectomes of the rat nervous system have been elaborated for the retrosplenial cortex (Sugar et al., 2011), nucleus of the solitary tract (Palombi et al., 2006), reticular formation (Humphries et al., 2006), hippocampus (Burns and Young, 2000), and the visual system (Burns, 1997), for an overview of connectome studies we refer to Sporns (2011), Schmitt and Eipert (2012). A connectome of the amygdala of the rat is reported here for the first time, even though, reviews of the hodology of the amygdala are numerous (Swanson and Petrovich, 1998; Pitkänen, 2000; Sah et al., 2003; de Olmos et al., 2004). In contrast to low-resolution, non-directed, and non-weighted connectomes derived from tractographic analysis of *in vivo* DTI measurements (Essen et al., 2012), the type of connectome described in the following is a high-resolution, directed, and weighted network where nodes are organized in a neuroontology containing functional, neurophysiological, and molecular biological information.

**Figure 1:**
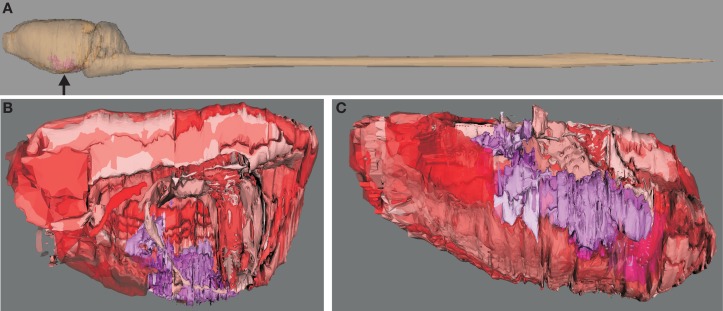
**Visualization of hierarchical regions of the rat central nervous system using *neuroVIISAS***. The subdivision of regions of the amygdala is based on the stereotactic atlas of Paxinos and Watson (2007). **(A)** Location of the amygdala (arrow) in the central nervous system of the rat. **(B)** The view from the midline into the right hemisphere shows the amygdala (magenta structures). Cortical regions are labeled with red tones [same orientation as shown in **(A)**]. This scheme of colors is consistent with the color mapping of the complete rat nervous system connectome where further colors are already assigned to other regions (see Materials and Methods). **(C)** The view from ventral (bottom) shows the rostrocaudal extension of the amygdala. The rough surfaces are caused by the intersection distance, serial sectioning, and small subregions of the amygdala.

Nodes of the network correspond to regions which are distinguishable in terms of cytoarchitecture. These regions can be related to each other with regard to hierarchical subdivisions. Since many possibilities exist to build such hierarchies of subdivisions of the amygdaloid complex that influence the analysis of its connectome, an overview will be provided in the following. The German translation *Mandelkern* of the term *amygdala* has been introduced by Burdach (1819–1826). A first topographic description of the amygdala was published by Meynert (1867). A nomenclature of subdivisions was introduced by Johnston (1923) and further developed by Pitkänen (2000) and de Olmos et al. (2004). Lists of terms of amygdala nuclei are contained in the stereotaxic atlases of Paxinos and Watson (1986) and Swanson (1992). A comprehensive comparison of nomenclatures was published by Price (1981) and Pitkänen (2000). The rat amygdaloid complex (amygdalar complex, amygdaloid body), amygdala in short, is a heterogeneous gray complex of 13 larger nuclear and cortical regions. This multinuclear complex is located in the depth of the anteromedial temporal lobe ventral to the lentiform nucleus (Figure 1). The topographical criteria proposed by Brockhaus (1938) to subdivide the amygdala into a superficial and a deep nuclear group has been adopted here as well as by other authors (Pitkänen, 2000; de Olmos et al., 2004) and will be described in detail in the next subsection.

In compliance with the structural diversity of the amygdala the various nuclear groups and subdivisions also differ functionally. Major outputs to functional systems generating experimental, motor, mnemonic, autonomic, or endocrine responses originate largely in different nuclei (Russchen, 1986). Four functional systems can be related to nuclear groups of the amygdala: the accessory olfactory, the main olfactory, the autonomic, and the frontotemporal system. The amygdala plays a crucial role in conditioned fear, anxiety, and attention. Especially the lateral, basolateral, and central nuclei of the amygdala build a functionally unified system necessary for the acquisition and expression of conditioned or instrumental fear learning and memory modulation (Davis, 1992). For example, the electric stimulation of the amygdala elicits a pattern of behaviors that mimic natural or conditioned fear (Rosen and Davis, 1990) and anxiolytic effects can be induced by applying benzodiazepine receptor antagonists in the basolateral nucleus (Hart et al., 2010). Distinct groups of neurons respond to (1) primary (unlearned) reinforcers like taste, (2) visual stimuli which previously have been paired with primary reinforcers like taste (positively reinforcing effects produced by novel stimuli independent of previous association of visual stimuli with primary reinforcement), and (3) responds to faces. Substantial evidence from both infra-human and human subject studies suggest that the sympathetic nervous system and the amygdala are crucial for the modulation of long-term memory storage for emotionally arousing events. The central and basolateral amygdaloid nuclei are involved in conditioned taste aversion, a unique type of innately predisposed (prepared) learning, in which the subject associates a taste with malaise over long delays (Yamamoto and Ueji, 2011). The consolidation of long-term explicit/declarative memory can be blocked after lesioning the basolateral amygdaloid nucleus (Gale et al., 2004).

Dysfunctions of the amygdala in humans have been reported in different neurological and psychiatric diseases like Klüver-Bucy-syndrome, Urbach-Wiethe-syndrome, temporal lobe epilepsy, Alzheimer disease, dementing disorders, and major depressive disorder. Generally, changes in emotionality and modulation of memory are attributed to lesions of the amygdala. A deficit in identifying fear in facial expressions of emotion while other expressions are intact was described by Adolphs et al. (1994) and Young et al. (1995). The importance of the amygdala for fear conditioning as well as promotion of effective memories (e.g., aversive conditioning) by arousing situations have been found in rodents (Chen et al., 2011, 2012). Neurotoxic lesion of the amygdala alters affective responses (Meunier et al., 1999). After lesion of the basolateral amygdaloid nucleus it was shown that the antidepressant fluoxetine has a positive effect on hippocampal cell survival (Castro et al., 2010). This emphasizes the fact that the amygdala may modulate the antidepressant action in hippocampal neurogenesis and its relation to depression-like behaviors. Furthermore, the basolateral amygdaloid nucleus seems to play an important role in the representation of the sensory features of motivationally significant events. Dwyer and Killcross (2006) demonstrated that animals with lesions of the basolateral amygdaloid nucleus can represent the sensory aspects of neutral events but not the sensory aspects of motivational events.

The connectional role of these regions which have been distinguished functionally can be determined by investigating the connectome of the amygdala in terms of graph theoretic and multivariate analysis. This contribution aims to establish and elucidate the connectome of the rat amygdala with a focus on intrinsic connectivity and emphasis on central, medial, lateral, and basal nuclear complexes.

## 2 Materials and Methods

Organization, visualization, and analysis of connectome data have been performed in *neuroVIISAS* (Schmitt and Eipert, 2012), a platform-independent generic framework developed in JAVA™. The *neuroVIISAS* installation package can be downloaded from http://neuroviisas.med.uni-rostock.de/index-Dateien/Page455.htm. All computations and visualization have been realized within the *neuroVIISAS* framework. The following pipeline describes the connectome analysis performed in this study:

1. Search, selection, and evaluation of tract-tracing publications
2. Defining the network of interest by selecting regions
3. Computing matrix representations
4. Global parameter computation
5. Local parameter computation
6. Motif analysis
7. Multivariate analysis
8. Vulnerability analysis
9. Extrinsic connectivity estimation

### 2.1 Nomenclature

**Figure 2:**
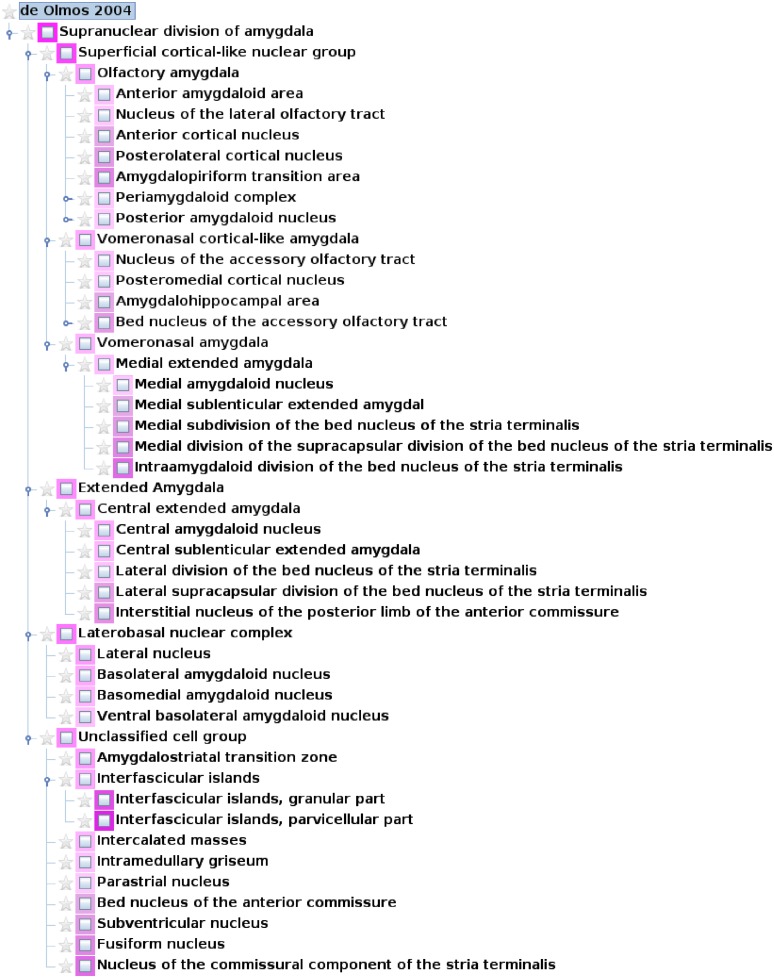
**A hierarchical nomenclature of subregions of the rat amygdala based on (de Olmos et al., 2004) has been implemented for the rat connectome project in *neuroVIISAS***. Only the top levels of the hierarchy are shown. Different intensities of magenta indicate different subregions.

The superficial cortical-like nuclear group consists of the anterior, posterolateral, posteromedial cortical amygdaloid nuclei, the amygdalohippocampal and amygdalopiriform transition area, the nucleus of the lateral olfactory tract, the nucleus of the accessory olfactory tract, the medial amygdaloid nucleus, and the anterior amygdaloid area. The nuclear groups that are located in the deep zones of the amygdaloid body are the ventral basolateral nucleus, the central amygdaloid nucleus, and the laterobasal nuclear complex as well as three divisions, the lateral, basolateral and basomedial nuclei, intercalated masses, amygdalostriatal zone, granular and parvicellular interfascicular islands, and intramedullary griseum. Additional groups are the extended amygdala (central and medial sublenticular extended amygdala, paraseptal lateral, and medial bed nuclei of the stria terminalis) and the unclassified cell group (part of “Other amygdala areas” in Table 1). This grouping of major nuclei and primary subdivisions is based on the work of de Olmos et al. (2004) and it is visualized in a hierarchy exported from *neuroVIISAS* (Figure 2). The hierarchical nomenclature of de Olmos et al. (2004) is based on cyto- and fibroarchitectonical criteria, ontogenetic, histo- and immunocytochemical, and hodological data. However, in the tract-tracing literature of the amygdala, much smaller subparts of nuclei and zones are described. In order to preserve this high-resolution spatial information, further subdivisions based on the nomenclature of de Olmos et al. (2004) have been taken into account. The de Olmos et al. (2004) nomenclature has been related to further (Paxinos and Watson, 1986; Swanson, 1992; Pitkänen, 2000; Price, 1981) terminologies (Table 1). In Table 1 major nuclear groups such as LA, Bnc, AB, Me, Ce, and AHi possess several subdivisions and are located at the 4th level of the amygdala hierarchy. The basal nucleus possesses a maximum of 89 subdivisions that were found in the amygdala tract-tracing literature. In the following, we refer only to an extended version of the (de Olmos et al., 2004) nomenclature obtained by subdividing the regions mentioned in tract-tracing studies.

**Table 1:** **Comparison of amygdala nomenclatures**.

Subdev.	Level	de Olmos et al. (2004) extended	Pitkänen (2000)	Paxinos and Watson (1986)	Swanson (1992)	Price (1981)
**DEEP NUCLEI**						
19	3	LA	L	La	LA *w.n.s*.	L *w.n.s*.
1	4	LaDL	Ldl	LaDL		
2	4	LaVL	Lvl	LaVL		
0	4	LaVM	Lm	LaVM		
89	4	*Bnc*	B	BL	BLA	B *w.n.s*.
4	5	Bmc	Bmc	BLA*	BLAa	
0	5	Bnci	Bi	*i.n*. BLA	*i.n*. BLAa	
7	5	Bpc	Bpc	BLP	BLAp	
9	4	*AB*	AB	BM	BMA	*w.n.s*.
0	5	ABm	ABmc	*i.n*. BMP	*i.n*. BMAp	
2	5	ABp	ABpc	BMP	BMAp	
**SUPERFICIAL NUCLEI**						
6	4	LOT	NLOT	LOT	NLOT	NLOT
4	4	BAOT	BAOT	BAOT	BA	BAOT
0	4	ACo	COa	ACo	COAa	
1	3	BMA		BMA	BMAa	
21	5	Me	M	Me	MEA	
0	6	MeRo	Mr	MeAV	MEAav	
2	6	MeCd	MeCd			
0	7	MeCd	Mcd	MeAD	MEAad	
0	7	MeCV	Mcv	MePV	MEApv	
0	7	MeC	Mc	MePD	MEApd	
3	4	PAC	PAC	Co	COA	PAC
3	4	PAC	PAC	PLCo	COApl	PAC = PLCo
0	5	PACm	PACm	*i.n*. PMCo	*i.n*. COApl	
0	5	PACs	PACs	*i.n*. PLCo	*i.n*. COApl	
1	4	COAp	COp	PMCo	COApm	Cop = PMCo
**OTHER AMYGDALOID AREAS**						
6	4	AA	AAA	AAD	*i.n*. AAA	AAA
28	4	Ce	CE	Ce	CEA	CE
2	6	Cec	CEc	*i.n*. CeLC	*i.n*. CEAc	
7	5	CeL	CE*_l_*	CeL	CEA*_l_*	
0	5	CeI	CEi	CeLCn	*i.n*. CEA*_l_*	
5	5	CeM	CEm	CeM	CEAm	
7	4	AHi	AHA	AHi		AHA
0	5	AHimp	AHAm	AHiPM	PA	
0	5	AHilp	AHAl	AHiAL	*i.n*. COApl	
4	3	IM	I	I	IA	I*

*The first 2 columns are related to the extended (de Olmos et al., 2004) nomenclature in *neuroVIISAS*. i.n., Included in; w.n.s., with no subdivisions*.

*BLA is the anterior basolateral nucleus (see also Abbreviations).

### 2.2 Data collection

Data retrieval was based on publications reporting tract-tracing studies of intrinsic and extrinsic amygdala connections using PubMed^1^, describing:

1. tract-tracing experiments using anterograde, retrograde, or bidirectional (where location of labeled perikaryons and axonal terminals are explicitly described) transport of substances
2. juvenile or adult rats
3. healthy, genetically un-altered and untreated control rats
4. results in English, German, or French

The search expression [amyg* *AND rat AND brain AND* (pathway* OR projection* OR afferent* OR efferent* OR connect*) *AND* (trace* OR tracing OR retrog* OR anterog*)] *NOT* (*virus OR viral*) yielded 487 papers, which were added to the connectome bibliography *references.bib* in JabRef^2^. This bibliographic information is also available online at the *neuroVIISAS* webpage. However, the PubMed search as described consider titles and abstracts of publications only. More connectivity data of the amygdala have been collected by evaluating tract-tracing publications of cortical areas and basal ganglia. At present, the whole rat connectome project contains data from 2100 tract-tracing publications. Papers not conforming with the criteria were marked in *references.bib* in order to exclude them automatically from search results. Intrinsic connections of the amygdala are described in 81, extrinsic input to the amygdala was found in 332, and extrinsic output from the amygdala in 337 publications.

The connectional information was gathered from these publications by the following criteria:

1. anterogradely labeled terminals and retrogradely marked perikaryons are clearly related to regions
2. locations of injection sites are described unambiguously
3. injection sites do not overlap with adjacent regions or fiber bundles
4. lesion and transsynaptic-tracing studies are excluded

The following data were retrieved from the publications and integrated in the rat connectome project of *neuroVIISAS*:

1. ipsilateral, contralateral, and bilateral connections
2. semiquantitative information that is often designated as the “weight” of a connection
3. tract-tracing substance
4. transport direction of tracer
5. case or individual rat where a connection has been observed
6. bibliographic details (stored in bibtex format) related to each connection
7. links to the pdf-documents of the tract-tracing publications

All information about connections is stored as objects in a zip-compressed project file that can be exported in spreadsheet format as well as in the owl ontology format of Protégé (Cimino and Zhu, 2006; Zhang et al., 2006; Poliakov et al., 2007; Musen et al., 2009). Before connectivity data were imported into *neuroVIISAS*, correctness was checked by at least two of the authors. The results of independent retrograde and anterograde experiments were integrated, such that the source of a connection, the target, its weight, and further connectivity attributes are available in a network representation that can be analyzed and visualized. All amygdala connections were added to the rat connectome data based on the hierarchy as shown in Figure 2. The interactive navigation, analysis, and visualization functions of *neuroVIISAS* allow a consistent and complete multi-level inspection of connectomes.

The subdivision of de Olmos et al. (2004) was used as an initial hierarchical nomenclature (Figure 2). This subdivision of regions of the amygdala is chiefly based upon cytoarchitectonic differences. However, it was extended based on descriptive subdivisions introduced in tract-tracing studies. The hierarchy consists of nodes (neuroanatomic regions) and relations whereby the relations in terms of an ontology belong to the type “is part.” Within the hierarchy, a root node (supranuclear division of the amygdala), nodes without further subdivisions (leafs), and nodes with further subdivisions exist. The sequence of hierarchically predefined leafs will be applied to matrix representations of connections and computations of further matrices to allow comparison. So far, 8 levels of subdivision of the amygdaloid complex are available in the connectome. Three regions have been mentioned in the literature that are possessing connections at the 8th level of subdivision of the amygdala: posterior basolateral nucleus parvicellular subdivision caudal part (Groenewegen et al., 1997), posterior basomedial nucleus medial half in the caudal part, and posterior basomedial nucleus lateral half in the rostral part (Kishi et al., 2006). The left- and right-hemispheric amygdala are connected by contralateral projections. The intrinsic connections of the left and right amygdala are the same. Since we have not found a lateralization of the amygdala connectivity, results of the left amygdala are presented.

The amygdala connectome is a partial connectome within the total connectome of the whole rat nervous system. Different ranges of colors are assigned to basic parts of the central nervous system, e.g., diencephalon, midbrain, cerebral cortex, oblongate medulla. Within this scheme of colors the amygdala subregions are assigned to shades of magenta to differentiate them from non-amygdala adjacent regions. The same colors of regions were consistently used in the hierarchy, 3D-visualization (Figure 1), matrices (Figure 4), and principal component analysis (Figure 9).

## 3 Results

In addition to the definitions, descriptions and interpretations of parameters and statistical procedures that are used in the results subsection, the supplement contains a complete list of definitions.

### 3.1 Global parameters of the intrinsic amygdala network

**Figure 3:**
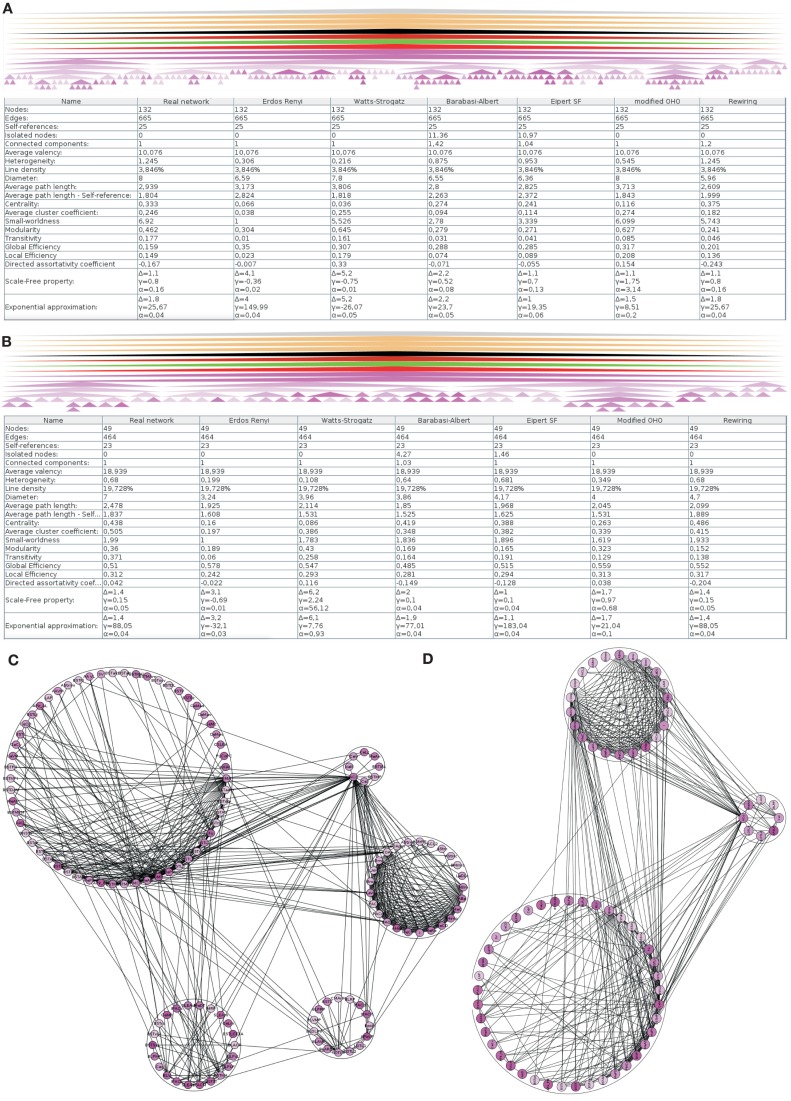
**Global analysis of the left intrinsic amygdala network applied to (A) all regions (132 regions and 665 edges) and (B) 49 regions that have at least one input and one output that are connected by 464 edges (condensed intrinsic amygdala network)**. The selected regions of the network are visualized by hierarchies of triangles (top panels). The bottom panels display the global network statistics. Both networks are simulated 1000 times by 6 different network generators and global parameters of 1000 simulations were averaged. The result of a modularity analysis is shown in **(C)** (all regions) and **(D)** (regions with at least one input and output). **(C)** Within the 5 modules the number of connections is larger than in between the modules. **(D)** The number of modules is reduced to 3 if only those regions are considered that have at least one input and one output (90° rotated view).

**Figure 4:**
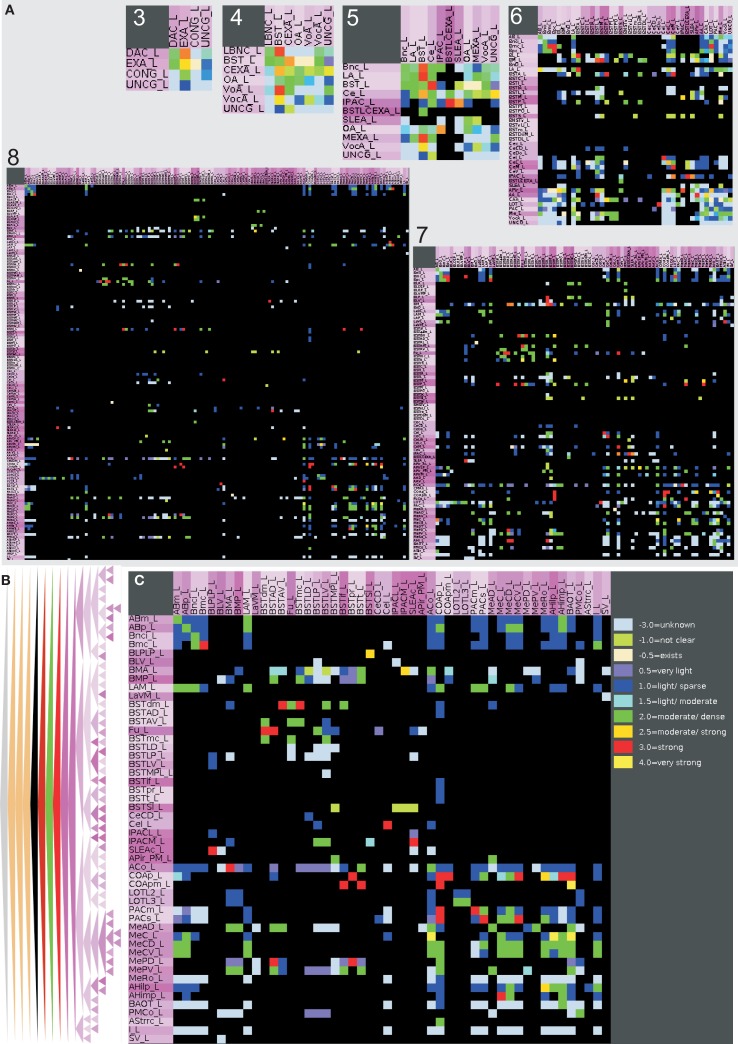
**Hierarchical connectomes allow the selection of different levels of subdivisions of regions. (A)** This multi-level representation is shown in the panel with gray background and applied to the left amygdala with 132 regions from level 3 to level 8. Sources or efferent regions are arranged in rows and target or afferent regions are arranged in columns. The sequence of regions is predefined by the sequence of leafs of the defined region hierarchy (see Materials and Methods). **(B)** Triangle representation of all regions that have at least one input and one output at level 8 and **(C)** the corresponding adjacency matrix describing 49 regions and 464 connections.

The hierarchy of regions of the left amygdala consists of 362 nodes. 279 of them do not posses further subregions. For 162 of these 279 regions of the left amygdala no intrinsic connections are documented. These 162 regions and, if needed, their parent regions were successively removed in order to build a network where each node has at least one connection. The resulting network contains 132 regions that are forming a single component connected by 665 edges (Figure 3A). A multi-level representation of the adjacency matrix is shown in Figure 4A. The final *condensed intrinsic network* contains only regions which have at least one input *and* one output (Figures 4B,C). This network has the benefit of being well-defined because regions where no connectivity data are available do not contribute to transmission of information in the network and should not influence global and local network parameters, limiting the value of any comparison with randomized networks. The condensed network contains 49 regions that are connected by 464 edges (Figure 3B). Both figures show the global network parameters and results of simulations [Erdös Rényi, Watts-Strogatz, Barabasi-Albert, Eipert (Eipert network), modified (directed graph) OHO (Ozik et al., 2004), and rewiring network generators (for details, see Schmitt and Eipert, 2012)] with the same number of nodes and edges as the real intrinsic left amygdala network. Simulations are used in order to compare the quantitative features of the real network with averaged parameters of different types of random networks that are simulated several times (see below). In the condensed network, the mean number of edges per node is 18.939 (average valency) and the line density (connections of the network divided by the number of all possible connections) 19.728%. The heterogeneity (standard deviation of valency divided by the average value) of 0.68 suggests that each node has a sum of inputs and outputs in the same range (Estrada, 2010). On average each node can be reached by using 2.478 edges (average path length). The average, number of links incident upon a node (centrality) is 0.438. A measure of how complete the neighborhood of a node is, is given by the average cluster coefficient (Rubinov and Sporns, 2010) and turns out be 0.505. Both global parameters are larger than the mean of 1000 Erdös Rényi simulations with the same number of nodes and edges hinting at a specific wiring structure. However, these parameters are more similar for the EN, modified OHO, and rewiring networks. In a small-world network most nodes are not neighbors of one another, however, most of them can be reached from every other node by a small number of edges. The small-worldness parameter (cluster coefficient of real and Erdös Rényi networks divided by average path lengths of real and Erdös Rényi networks) of 1.99 is the largest, compared to simulated networks (Humphries et al., 2006; Humphries and Gurney, 2008). Hence, the intrinsic amygdala network has a small-world property. Interestingly, the average path length of 2.478 is the largest compared to all simulations. A small average path length and a large average cluster coefficient hint at a small-world network, consistent with the small-worldness parameter. The condensed amygdala network has a relatively small delta error (mean deviation between the data and the approximation of the power law distribution multiplied by 100) of 1.4 in comparison with a power law distribution, pointing to a scale-free property. The modularity Newman (2004) of 0.36 is relatively small, indicating dense connections between modules of the intrinsic amygdala network (Figures 3C,D).

### 3.2 Network

**Figure 5:**
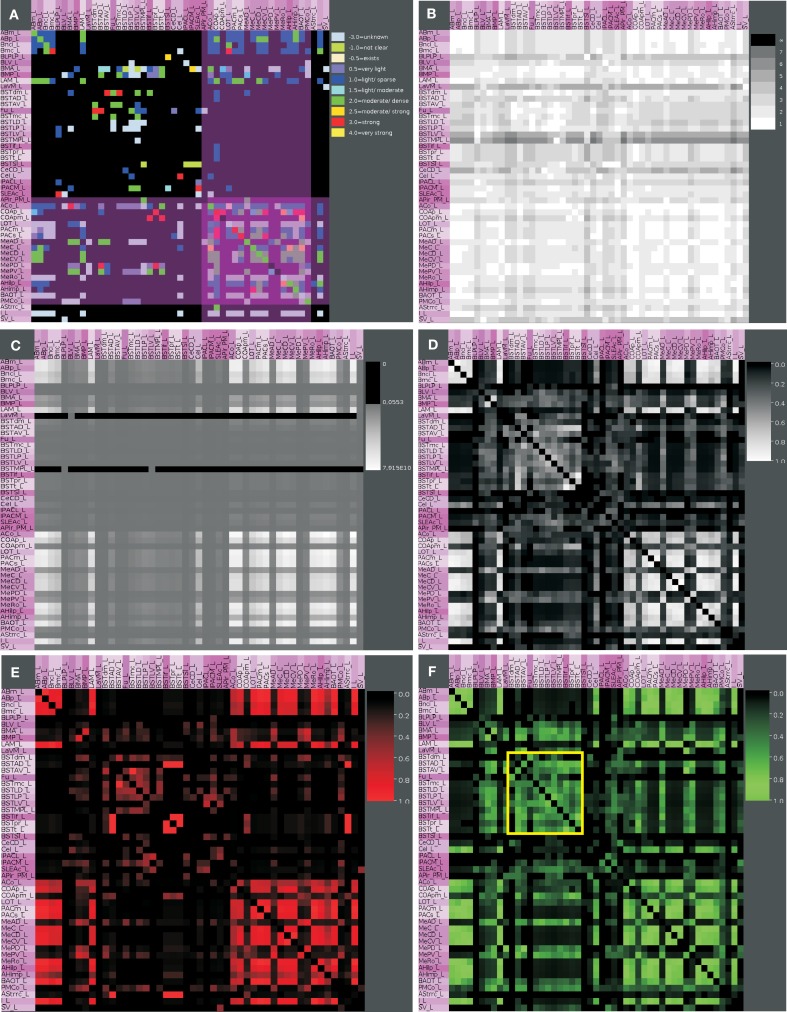
**Connectivity matrices of the condensed intrinsic left amygdala network**. **(A)** Adjacency matrix with highlighted regions (transparent magenta) of the superficial amygdaloid cortex. Ten connectivity weights are color-coded. **(B)** Distance matrix. Gray levels 1–7 indicate the smallest number of edges between regions. Light gray values indicate short distances. **(C)** Communicability matrix. The gray scale codes values between 0.0553 and 7.915E10. Large values indicate (e.g., ACo) that many short shortest paths between a pair of regions exist. **(D)** Connectivity matching matrix for inputs and outputs. The gray level scale is coding values between 0 and 1. Large values indicate similar input and output connections of two regions. **(E)** Connectivity matching matrix for inputs. Red shades are coding similar inputs of two regions. **(F)** Connectivity matching matrix for outputs. Green shades are coding the similar outputs of two regions. The yellow square indicates the regions of the bed nucleus of the stria terminalis.

The rows of the adjacency matrix indicate sources, perikaryon locations or efferents, and the columns are indicating targets, axonal terminal locations, or afferents. The adjacency matrix shows a cluster of dense interconnections of the subregions of the superficial amygdaloid cortex (superficial cortical-like nuclear group; Figure 5A). The different subdivisions of the amygdaloid nucleus are subregions of the superficial amygdaloid cortex and possess numerous outputs (103) to anterior basomedial and accessory basal nuclei. The cluster of strong local connectivity within subregions of the superficial amygdaloid cortex can be found again in the distance matrix where most of these connections turn out to use only one edge (Figure 5B) which means that most of these regions are directly interconnected. The ventromedial part of the lateral nucleus, the bed nucleus of the stria terminalis lateral division ventral part, and the bed nucleus of the stria terminalis medial division posterolateral part attract attention due to many long distances indicated by dark gray values in the correspondent rows. The bed nucleus of the stria terminalis medial division posterolateral part and the ventromedial part of the lateral nucleus show low communicability values (Estrada and Hatano, 2008; only few shortest paths between a pair of connected regions exist), whereby the bed nucleus of the stria terminalis lateral division ventral part has similar values as neighboring nuclei (Figure 5B). The subregions of the superficial amygdaloid cortex are forming a cluster with large communicability values, hence, many shortest paths between pairs of connected regions exist. The matrix of connectivity in degree matchings (Sporns, 2002) shows a new set of regions that have quite similar input patterns of connectivity. These subregions belong to the bed nucleus of the stria terminalis and are located within the upper left part of the matrix indicated by a yellow square (Figure 5F). The subregions of the bed nucleus of the stria terminalis have lower connectivity output matching values (Sporns, 2002; Figure 5E) than input values which indicates more similarity of input connections *to* than output connections *from* BST-subregions. In the connectivity input and output matching matrix, the larger values of the subregions of the bed nucleus of the stria terminalis are highlighted again and the second cluster of the superficial amygdaloid nucleus is also visible (Figure 5D). The large input connectivity matching means that pairs of regions have similar afferents. Because the same regions have low output connectivity matching values, processed information of them is distributed divergently to target regions.

### 3.3 Local network parameters

**Figure 6:**
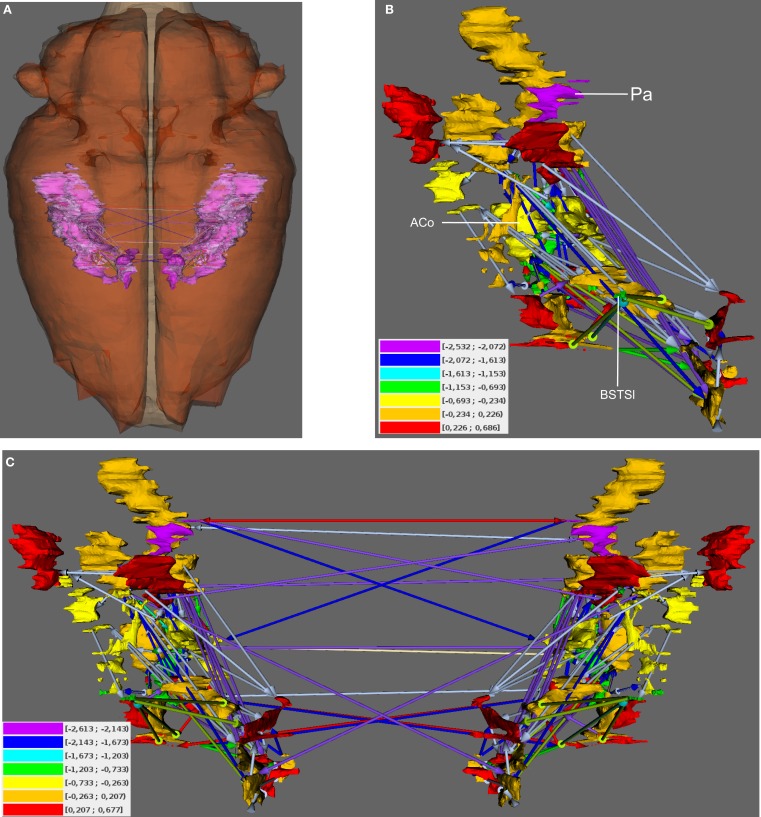
**Visualization of Shapley rates in 3D-expansion view of the unilateral and bilateral amygdala**. **(A)** View from dorsal. The left- and right-hemispheric amygdala are visible through the transparent pars cranialis of the central nervous system. **(B)** Unilateral amygdala with regions that have the lowest Shapley rates (see text) and color-coded weights of connections (color-codes of connections are the same as in Figure 5). Color-coded Shapley rates are assigned to regions. **(C)** Bilateral amygdala with ipsilateral and contralateral connections.

Local network analysis using the same set of regions as in the global network analysis reveals the impact of single regions with regard to connectivity and flow of information. Most of the 30 local network parameters calculated by *neuroVIISAS* (Table 2) are strongly correlated with the *degree all* parameter [the *degree* of a node (vertex) is the number of incoming (afferent) and outgoing (efferent) edges (connections)]. If incoming connections are differentiated from outgoing connections, then the terms *indegrees* (in) and *outdegrees* (out) are used (Sporns 2002). The anterior cortical amygdaloid nucleus has the largest degree all (55) followed by the posterior amygdaloid nucleus (41) and the bed nucleus of the accessory olfactory tract (33). However, the Shapley rate (a low Shapley rate indicates a large impact of a node in a network; Kötter et al., 2007) of the posterior amygdaloid nucleus is smaller (−2.532) than that of the anterior cortical amygdaloid nucleus (−0.759). Interestingly, the Shapley rate of the supracapsular bed nucleus of the stria terminalis lateral part is the second smallest (−0.91), although only having 13 inputs and outputs. The Shapley rates are visualized in the 3D-atlas using a multiaxes extension (Figure 6).

**Table 2:** **Local parameters of the condensed amygdala network sorted by the *degree all* parameter**.

	DGa	DGo	DGi	Katz	LC	Ecco	Ecci	CCo	CCi	CCa	CC2	ADG	VC	L	Co	Ci	BC	EC	St	Shapley	Zo	Zi	Za	PCo	PCi	PCa	Ro	Ri	Ceno	Ceni
ACo	55	32	23	3362.98	1	3	5	0.29	0.43	0.26	0.08	20.94	0.5	0.83	0.75	0.53	869.29	1.000000	1840	−0.79	0.75	1.04	0.96	0.55	0.48	0.53	6.39	6.12	14	3
COAp	41	16	25	3376.27	1	4	6	0.63	0.36	0.37	0.19	23.19	0.51	0.79	0.52	0.52	376.99	0.786587	928	−2.51	1.73	1.61	1.67	0.32	0.55	0.48	5.84	6.06	−27	−3
BAOT	37	18	19	3024.39	1	4	6	0.72	0.52	0.49	0.08	27.17	0.35	0.78	0.53	0.49	175.4	0.926399	475	−0.34	0.75	−0.57	0.37	0.2	0.52	0.39	5.88	5.98	−25	−7
PACs	33	18	15	3143.98	1	4	6	0.71	0.9	0.71	0.21	28.67	0.37	0.85	0.53	0.44	38.4	0.919279	116	−0.21	0.75	0.51	0.76	0.2	0.12	0.17	5.88	5.73	−25	−19
I	33	17	16	3362.76	1	4	6	0.8	0.87	0.8	0.19	30.18	0.29	0.85	0.53	0.45	11.86	0.919269	115	−0.17	0.75	1.04	0.96	0.11	0.12	0.11	5.86	5.76	−26	−18
BMA	32	21	11	385.8	2	3	5	0.2	0.33	0.18	0.36	14.21	0.8	0.55	0.62	0.47	322.15	0.214069	813	−0.48	2.8	1.86	3.06	0.33	0.17	0.28	6.12	5.86	−14	−14
ABm	31	16	15	3143.98	1	4	6	0.88	0.9	0.88	0.17	31.56	0.24	0.83	0.52	0.44	4.37	0.914638	47	−0.13	0.5	0.51	0.57	0.12	0.12	0.12	5.84	5.73	−27	−19
ABp	31	16	15	3225.51	1	4	6	0.88	0.9	0.88	0.17	31.56	0.24	0.83	0.52	0.44	8.93	0.914638	91	−0.16	0.5	0.51	0.57	0.12	0.12	0.12	5.84	5.73	−27	−19
MeRo	31	17	14	3023.02	1	4	6	0.81	0.91	0.81	0.19	30.29	0.29	0.85	0.53	0.44	10.77	0.919269	106	−0.14	0.75	−0.03	0.57	0.11	0.13	0.12	5.86	5.71	−26	−20
PACm	30	17	13	2793.45	1	4	6	0.81	0.94	0.81	0.19	30.35	0.29	0.85	0.53	0.43	5.64	0.919269	54	−0.1	0.75	−0.57	0.37	0.11	0.14	0.12	5.86	5.69	−26	−21
MeCD	30	14	16	3362.76	1	4	6	0.92	0.88	0.88	0.17	31.63	0.24	0.83	0.51	0.45	6.31	0.820732	68	−0.13	−0.02	1.04	0.37	0.13	0.12	0.12	5.8	5.76	−29	−18
AHilp	30	15	15	3143.98	1	4	6	0.8	0.9	0.78	0.19	29.88	0.34	0.83	0.52	0.44	51.12	0.820169	104	−0.43	−0.02	0.51	0.17	0.23	0.12	0.18	5.82	5.73	−28	−19
MeCV	29	14	15	3143.98	1	4	6	0.93	0.9	0.88	0.17	31.69	0.24	0.83	0.51	0.44	1.95	0.820732	23	−0.09	−0.02	0.51	0.17	0.13	0.12	0.13	5.8	5.73	−29	−19
MeC	27	14	13	2885.77	1	4	6	0.81	0.96	0.83	0.17	30.88	0.29	0.78	0.51	0.43	8.43	0.758913	85	−0.08	0.24	−0.57	−0.02	0	0.14	0.07	5.78	5.69	−30	−21
LAM	26	13	13	2719.76	1	4	6	0.89	0.92	0.9	0.17	31.88	0.23	0.82	0.51	0.4	0.92	0.745062	10	−0.07	−0.27	−0.03	−0.22	0.14	0	0.07	5.78	5.53	−30	−29
Bmc	25	15	10	2108.64	1	4	6	0.7	0.94	0.74	0.16	30.12	0.31	0.74	0.52	0.4	6.23	0.757125	17	−0.01	−0.02	−1.64	−0.61	0.24	0	0.15	5.82	5.47	−28	−32
Bnci	24	12	12	2500.98	1	4	6	0.89	0.92	0.9	0.17	32	0.23	0.82	0.5	0.4	0.53	0.685212	6	−0.03	−0.53	−0.57	−0.61	0.15	0	0.08	5.76	5.51	−31	−30
BMP	23	15	8	346.31	2	3	6	0.28	0.54	0.25	0.29	15.06	0.79	0.5	0.57	0.42	33.26	0.142449	126	0.11	1.66	0.81	1.68	0.34	0.22	0.3	6	5.61	−20	−26
AHimp	23	7	16	3362.76	1	5	6	0.98	0.91	0.91	0.17	32.06	0.23	0.82	0.38	0.45	0.95	0.421031	11	0.04	−1.8	1.04	−1.01	0.24	0.12	0.16	5.12	5.76	−38	−18
MeAD	22	15	7	345.54	1	3	6	0.31	0.43	0.3	0.19	20.06	0.69	0.41	0.57	0.43	150.68	0.256080	425	−0.38	1.66	0.47	1.5	0.34	0.24	0.31	5.98	5.65	−20	−23
MePV	21	17	4	309.5	2	3	6	0.31	1	0.31	0.2	19.59	0.7	0.42	0.58	0.4	36.34	0.245812	137	0.15	2.12	−0.57	1.33	0.3	0.38	0.32	6.02	5.47	−19	−33
PMCo	18	8	10	482.28	1	3	6	0.41	0.32	0.29	0.21	18.31	0.5	0.36	0.52	0.44	127.36	0.109455	310	0.02	0.53	1.16	0.98	0.22	0.32	0.28	5.84	5.71	−27	−22
BSTLP	17	6	11	538.41	1	4	5	0.3	0.37	0.32	0.22	17.86	0.7	0.41	0.44	0.48	151	0.021129	481	0.01	0.3	1.51	0.98	0	0.3	0.21	5.47	5.92	−31	−15
BSTLV	15	1	14	492.36	4	6	4	0	0.38	0.34	0.23	17.47	0.7	0.45	0.21	0.51	75.96	0.000003	181	0.34	−0.83	2.9	0.81	0	0.13	0.12	3.16	6.04	−46	−14
MePD	15	12	3	72.93	2	3	6	0.2	1	0.3	0.24	17.57	0.67	0.36	0.53	0.33	4.39	0.155683	22	0.37	0.98	−0.57	0.47	0.4	0	0.34	5.88	4.94	−26	−45
Fu	13	8	5	94.1	2	4	6	0.09	0.35	0.15	0.26	9.73	0.4	0.33	0.41	0.35	77.7	0.016061	213	0.05	0.76	0.12	0.64	0	0	0	5.31	5.12	−33	−40
BSTdm	11	8	3	33.12	2	4	7	0.29	0.5	0.29	0.21	18.5	0.62	0.2	0.49	0.32	89.59	0.087119	260	0.14	0.53	−0.57	0.12	0.22	0	0.17	5.73	4.86	−27	−46
BSTLD	11	4	7	394.2	2	5	6	0.5	0.55	0.42	0.2	20.7	0.66	0.29	0.32	0.43	59.9	0.002426	201	0.01	−0.15	0.47	0.12	0	0.24	0.17	4.71	5.65	−38	−26
BSTMPL	10	1	9	349.66	4	7	5	0	0.32	0.32	0.19	19.4	0.73	0.24	0.18	0.47	2.89	0.000002	8	0.69	−0.83	1.16	−0.05	0	0.2	0.18	2.27	5.88	−47	−17
CeI	10	2	8	1649.67	1	3	6	0.5	0.93	0.74	0.17	32	0.31	0.27	0.45	0.39	8.83	0.071119	48	0.42	−3.08	−2.72	−3.37	0.5	0	0.18	5.53	5.43	−42	−34
SLEAc	10	3	7	310.08	1	3	4	0.17	0.31	0.26	0.22	16.33	0.98	0.24	0.46	0.46	209.57	0.070288	432	0.1	−0.6	0.47	−0.23	0.44	0.24	0.32	5.59	5.84	−39	−14
BSTAD	9	1	8	578.12	2	5	6	0	0.54	0.54	0.17	27.75	0.48	0.2	0.35	0.45	10.56	0.051212	47	0.5	−1.06	0.47	−0.57	0	0.41	0.49	4.9	5.8	−47	−17
BSTAV	9	4	5	80.67	2	4	6	0.67	0.6	0.43	0.13	17.13	0.42	0.29	0.37	0.34	12.46	0.007160	34	0.38	−0.15	0.12	−0.05	0	0	0	5.06	5.04	−37	−41
BLV	8	3	5	316.06	2	4	5	0.17	0.3	0.26	0.22	18.57	0.88	0.16	0.37	0.43	137.63	0.008807	268	−0.48	−0.38	−0.23	−0.4	0	0.32	0.22	5.08	5.69	−40	−26
BSTmc	8	3	5	293.57	2	4	6	0.67	0.5	0.45	0.2	22.57	0.67	0.19	0.36	0.4	40.18	0.006722	95	0.39	−0.38	−0.23	−0.4	0	0.32	0.22	5.04	5.53	−38	−31
BSTif	8	1	7	351.09	3	5	6	0	0.48	0.48	0.19	25.75	0.58	0.2	0.35	0.42	0.39	0.051212	4	0.57	−1.06	0.12	−0.75	0	0.45	0.53	4.92	5.61	−47	−27
COApm	8	4	4	520.22	2	5	6	0.33	0.67	0.5	0.19	29	0.56	0.16	0.37	0.42	6.81	0.118195	19	0.39	−0.58	−0.23	−0.33	0.63	0.63	0.63	5.1	5.59	−42	−27
BSTt	7	1	6	348.6	3	5	6	0	0.63	0.6	0.19	27.86	0.54	0.19	0.35	0.4	0.39	0.051212	4	0.57	−1.06	−0.23	−0.92	0	0.5	0.57	4.92	5.53	−47	−31
IPACM	7	5	2	27.82	2	4	5	0.35	0	0.35	0.16	16	0.55	0.19	0.42	0.34	75.17	0.020302	163	0.15	0.08	−0.92	−0.4	0	0	0	5.41	5.06	−35	−39
BSTSl	6	4	2	8.95	2	4	5	0.17	0	0.2	0.37	7.2	0.34	0.31	0.36	0.28	145.33	0.006200	224	−0.91	−0.15	−0.92	−0.57	0	0	0	4.98	4.49	−39	−43
LOTL2	6	5	1	0.2	1	3	1	0.7	0	0.7	0.22	26.8	0.61	0.2	0.48	1	0	0.109761	0	0.5	−0.15	−1.27	−0.75	0.32	0	0.28	5.92	0.14	−35	−48
LOTL3	6	5	1	0.2	1	3	1	0.7	0	0.7	0.22	26.8	0.61	0.2	0.48	1	0	0.109761	0	0.5	−0.15	−1.27	−0.75	0.32	0	0.28	5.92	0.14	−35	−48
BLPLP	5	1	4	95.57	3	5	4	0	0.25	0.25	0.3	10.4	0.48	0.21	0.27	0.37	131.52	0.000400	291	−0.36	−0.83	−0.23	−0.75	0	0	0	4.06	5.33	−46	−37
BSTpr	5	1	4	291.47	2	5	6	0	0.5	0.5	0.21	27.75	0.35	0.1	0.35	0.4	0.39	0.051212	4	0.5	−0.58	−0.23	−0.33	0	0.38	0.48	4.9	5.51	−47	−28
LaVM	4	1	3	51.17	5	6	7	0	0.67	0.33	0.23	15.25	0.5	0.14	0.22	0.31	2	0.000030	4	0.61	−0.83	−0.57	−0.92	0	0	0	3.31	4.8	−46	−46
IPACL	4	2	2	22.97	3	4	5	0.5	0	0.33	0.36	7.25	0.26	0.32	0.33	0.32	5.93	0.004604	14	0.47	−0.6	−0.92	−0.92	0	0	0	4.73	4.92	−44	−39
APirPM	4	2	2	30.94	2	4	7	0.5	0	0.33	0.25	15	0.34	0.14	0.37	0.32	3.06	0.016678	11	0.45	−0.6	−0.92	−0.92	0	0	0	5.06	4.86	−46	−47
CeCD	3	1	2	225.51	3	6	7	0	0	0.17	0.33	19	0.52	0.07	0.25	0.36	1.87	0.000100	5	0.58	−0.83	−1.27	−1.26	0	0.5	0.44	3.8	5.18	−46	−34
SV	3	1	2	30.75	4	5	6	0	0	0	0.18	14.67	0.84	0.07	0.27	0.33	47.55	0.000600	92	0.28	−0.83	−0.92	−1.09	0	0	0	4.18	5	−46	−46
AStrrc	2	1	1	218.78	3	5	7	0	0	1	0.39	35.5	0.15	0.06	0.35	0.31	0	0.051212	0	0.67	−0.58	−1.15	−1	0	0	0.5	4.92	4.78	−47	−49

*For long names of region abbreviations, see abbreviation list. Parameter definitions are explained in the supplement as well as in reviews (Kötter, 2002; Rubinov and Sporns, 2010; Sporns, 2011). DGa, degree all; DGo, degree out; DGi, degree in; Katz, Katz index; LC, distance to the region itself; Ecco, eccentricity out; Ecci, eccentricity in; CCo, cluster coefficient out; CCi, cluster coefficient in; CCa, cluster coefficient in and out; CC2, cluster coefficient of 2nd neighbors; ADG, average neighbor degree; VC, variation coefficient of neighbor degree (Echtermeyer et al., 2011); L, locality; Co, closeness centrality out; Ci, closeness centrality in; BC, betweenness centrality; EC, eigenvector centrality; St, number of shortest paths; Shapley, Shapley rating; Zo, z-score out; Zi, z-score in; Za, z-score in and out; PCo, participation coefficient out; PCi, participation coefficient in; PCa, participation coefficient in and out; Ro, radiality out; Ri, radiality in; Ceno, centroid out; Ceni, centroid in*.

Most cyclic pathways from the anterior cortical amygdaloid nucleus back to it are possible via 21 different 2-edge-cycles, followed by the intercalated nuclei (16 different 2-edge-cycles) and the accessory basal nucleus parvicellular division, periamygdaloid complex sulcal division, and accessory basal nucleus magnocellular division (15 different 2-edge-cycles).

As described in the introduction, the lateral (LA), the basolateral (BL), and the central amygdaloid nucleus (CE) are frequently analyzed in behavioral, neurophysiologic, and pharmacologic studies. In addition, the medial amygdaloid nucleus (ME) is considered as an important entity (Aggleton 2000; Pitkänen 2000) of amygdaloid circuits and has been analyzed in the context of LA, BL, and CE. In the hierarchy that is used here, the LA and BL are child nodes of the laterobasal nuclear complex, CE is a subregion of the central extended amygdala, and ME is a subregion of the medial extended amygdala. They are not directly visible because some of their subregions are expanded. However, by reducing some branches of the hierarchy, they become leafs of the hierarchy (26 regions, 350 connections; not shown here) and local parameters of these regions can be computed. The CE has the smallest Shapley rate and most inputs and outputs (indegree: 22, outdegree: 22) indicate its large impact of the intrinsic amygdala connectome. ME (outdegree: 20, indegree: 21) has the second lowest Shapley rate of −0.076. The Shapley rate of BL is −0.003 (outdegree: 14, indegree: 15) and that of LA is −0.029 (outdegree: 17, indegree: 17). With regard to the other 24 regions, ME has the 3rd, BL the 9th, and LA the 10th smallest Shapley value. Hence, their intrinsic impact of BL and LA are considerably smaller than those of CE and ME. In the network where CE, ME, LA, and BL are leafs, most cyclic pathways are passing CE (2 node cycles: 19 passages, 3-node passages: 254) and ME (2 node cycles: 18 passages, 3-node passages: 247; LA 2 node cycles: 15, 3-node cycles: 198; BL 2 node cycles: 10, 3-node cycles: 121).

The sum of extrinsic contralateral and ipsilateral input of CE is 506 followed by BL (286), LA (230), and ME (216). The extrinsic output of CE is 380, of BL is 174, of LA is 109, and ME has the 2nd largest: 207. These four regions receive most extrinsic inputs among all regions of the 26 node hierarchy.

### 3.4 Motifs and circuits

**Figure 7:**
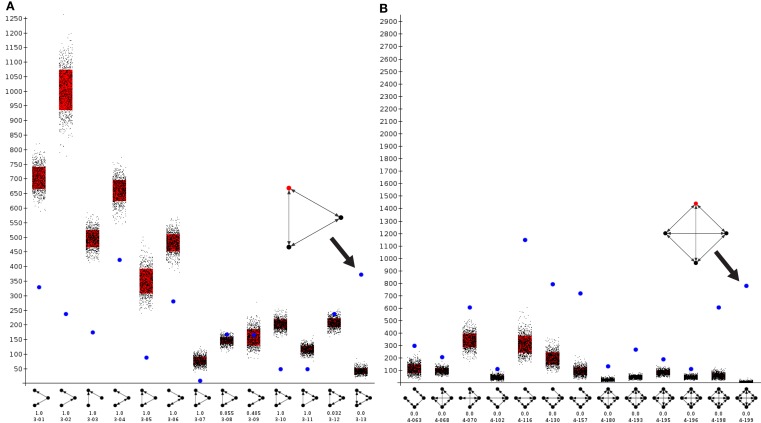
**Motif analysis of the condensed intrinsic amygdala network**. Frequency of motifs in the intrinsic amygdala network is indicated by a blue dot. Frequencies of motifs in rewiring randomizations are indicated by black points. Red bars show the standard deviation of motif frequencies. **(A)** 3-Node directed motif analysis of 13 subgraphs. The abundant fully reciprocal motif (e.g., 3-13, where 3 is the number of nodes of this motif and 13 is the identification number of a this motif) is indicated by an arrow and magnified. **(B)** 4-Node directed motif analysis. Those subgraphs that were significantly more frequent in the real network are shown.

Motif analysis for directed 3-node and 4-node motifs revealed a significantly more frequent expression of completely reciprocal motifs (all edges of a motif are reciprocal) than in a random network (Figure 7A). A maximum of 13 directed motifs that consist of 3-nodes and 199 that consist of 4 nodes can be constructed. The frequency of all motifs were determined by a paralleled (using n cores in parallel) isomorphism search (Schmitt 2012). The connections were randomized 1000 times (1000 networks with the same number of nodes and edges as the condensed intrinsic amygdala network were computed) by the rewiring simulation, the motif frequencies were determined and visualized by black dots in the motif-diagram. Blue dots that are above or beyond the black point clouds indicate a significant larger or lower frequency of occurrence in the real amygdala network in comparison to rewired networks. The first 6 motifs can be considered as variations of convergent and divergent motifs. These are all less frequent in the real than in the rewired networks. The cyclic motifs (3-07, 3-10) are also less frequent in the real amygdala network (Table 3). The completely reciprocal motif (3-13) is much more abundant in the real than in rewired networks (Figure 7A, arrow). The 4-node subgraph analysis shows similar results as the 3-node subgraph analysis (Figure 7B). Variations of divergent and convergent 4-node motifs (data not shown) as well as the completely reciprocal motifs are much more frequent in the rewired than in the real amygdala network. In Figure 7B only those motifs are shown for which p = 0 (p = 0 indicates motifs that are significant more frequent in a real network, p = 1 indicates motifs that are significant more frequent in a random network; Table 4). In the motif 4-116 the motif 3-13 is included and it occurs most frequently.

**Table 3:** **All 13 3-node motifs and resampling calculations based on 1000 rewiring randomizations of 49 regions and 464 connections**.

MN	*N*	*E*	*f*1	*f*2	*f*3	*p*	*z*	$\bar{x}$	σ
3-01	3	2	328	51	10	1	−9.983	701.373	37.39748
3-02	3	2	235	41	11	1	−11.046	1003.56	69.57714
3-03	3	2	173	43	10	1	−10.978	494.202	29.25675
3-04	3	3	421	48	10	1	−6.663	658.326	35.61592
3-05	3	3	87	24	10	1	−6.225	347.777	41.89099
3-06	3	3	279	41	7	1	−6.669	479.78	30.10242
3-07	3	3	7	4	4	1	−4.933	75.619	13.90999
3-08	3	4	165	35	9	0.055	1.688	145.338	11.6419
3-09	3	4	163	18	5	0.405	0.237	156.479	27.45046
3-10	3	4	47	16	6	1	−9.607	200.794	16.00711
3-11	3	4	46	16	5	1	−5.845	115.055	11.81347
3-12	3	5	235	36	7	0.032	1.873	205.676	15.65589
3-13	3	6	371	32	6	0	37.688	41.056	8.75459

*MN, motif number (corresponding motif type is shown in Figure 7); *N*, number of motif nodes; *E*, number of motif edges; *f*1, motif frequency with repeated usage of edges; *f*2, motif frequency without multiple usage of edges; *f*3, motif frequency without multiple usage of nodes; p, probability that motif frequency in random graph is greater than in the real network; *z*:(*f*1 – $\bar{x}$)/σ, $\bar{x}$: average frequency of a motif in network randomizations; σ, standard deviation*.

**Table 4:** **13 Significant 4-node motifs from a total of 199 4-node motifs and 1000 rewiring randomizations of 49 regions and 464 connections**.

MN	*N*	*E*	*f*1	*f*2	*f*3	*p*	*z*	$\bar{x}$	σ
4-063	4	5	295	14	4	0	5.074	111.351	36.18718
4-068	4	6	202	13	4	0	5.591	96.261	18.90949
4-070	4	6	604	12	4	0	4.742	336.156	56.47799
4-102	4	6	110	8	4	0	4.941	42.609	13.63694
4-116	4	7	1143	19	5	0	11.981	309.257	69.58759
4-130	4	7	789	16	4	0	12.175	193.728	48.8891
4-157	4	8	717	11	4	0	21.392	92.144	29.20872
4-180	4	9	128	9	4	0	13.236	23.404	7.90182
4-193	4	10	266	13	3	0	21.570	45.253	10.23391
4-195	4	10	188	10	3	0	6.6218	82.028	16.00348
4-196	4	10	108	8	4	0	11.981	45.77	10.66729
4-198	4	11	604	13	4	0	30.638	56.922	17.85587
4-199	4	12	777	14	5	0	166.413	7.047	4.62675

*See Table 3 for further details*.

The particular regions that are part of the completely reciprocal 3-13 motif are the anterior cortical amygdaloid nucleus (90 times), the intercalated nuclei of the amygdala (90), the periamygdaloid complex sulcal division (84), and accessory basal nuclei (83). The central division of the sublenticular extended amygdala (3), the ventral basolateral nucleus (2), and the posterior basolateral nucleus lateral part (2) occur in the cyclic 3-07 motif with almost no reciprocal connections. The frequencies of regions in the completely reciprocal 4-199 motif is similar to the case of motif 3-13: anterior cortical amygdaloid nucleus (258), intercalated nuclei of the amygdala (268), periamygdaloid complex sulcal division (254), and accessory basal nuclei (254). In contrast to the motif 3-13 region, participation frequencies of the intercalated nuclei of the amygdala are higher than the anterior cortical amygdaloid nucleus for motif 4-199.

The four regions LA, BL, CE, and ME have been analyzed with regard to local parameters and extrinsic connectivity (see above). The motif-search revealed that ME is part of the 3-13 motif in 90 cases, CE (86), LA (69), and BL (19). Interestingly, BL is most frequently a member in the 3-04 motif with one reciprocal edge and a single directed edge (CE: 31, LA: 23, ME: 20). The motif 3-09 with two reciprocal edges has another frequency distribution with regard to these regions (CE: 47, ME: 40, LA: 32, BL: 25).

**Figure 8:**
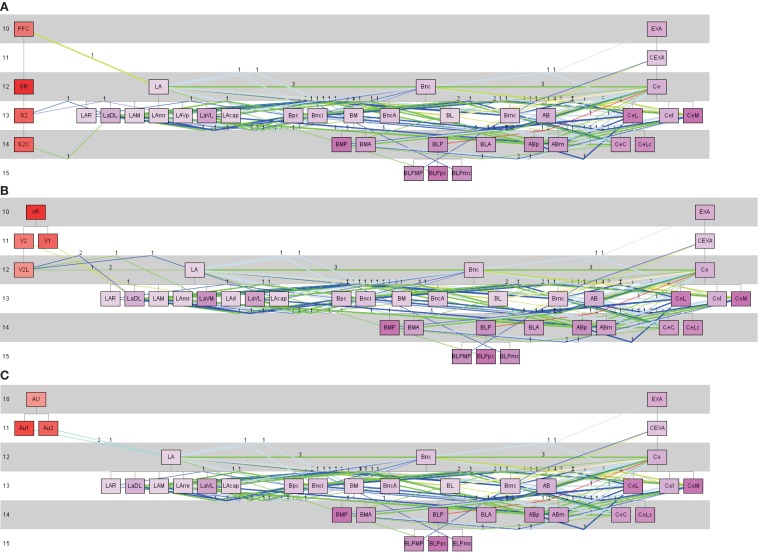
**Pathway analysis of somatosensory, visual and auditory regions of the cerebral cortex and the lateral, basal, and central nuclear complexes of the amygdala**. **(A)** Somatosensory, **(B)** visual, and **(C)** auditory cortical regions are defined as source regions of the projection to the lateral (LA) nuclear complex of the amygdala. All subregions of the LA are taken into account as putative target regions. From these LA-target regions connections to the basal nuclear complex (including BL) where searched. From the basal nuclear complex, connections were chosen which project to CE subregions. The colors of lines corresponds to the weight code in Figure 4. Thick lines indicate reciprocal connections and the numbers indicate the number of studies that have documented a particular connection. In the left part are the source regions displayed, then the LA subtree regions are following, then the basal nuclear complex subtree regions, and on the right the subregions of the CE subtree. The numbers at the left part of the pathway-diagrams are indicating hierarchical levels.

LA receives sensory inputs (Kim and Jung, 2006; Ciocchi et al., 2010; Haubensak et al., 2010; Tye and Deisseroth, 2012). Some of these sensory inputs originate in the somatosensory, auditory, and visual cortical regions. Typically LA projects to the basal nuclear complex which projects to CE (Ciocchi et al., 2010; Haubensak et al., 2010; Tye and Deisseroth, 2012). In terms of behavior this pathway is interpreted as the fear conditioning pathway (Kim and Jung, 2006; Ciocchi et al., 2010; Haubensak et al., 2010; Tye and Deisseroth, 2012). The rat connectome was analyzed by a constrained search using the pathway analysis approach of *neuroVIISAS* to detect pathways through LA → Bnc → CE. CE seems to be necessary for the fear response. As shown in Figure 8, the pathways from somatosensory, visual, and auditory regions to LA, from LA to Bnc and from Bnc to the output complex, CE can be reconstructed using a transhierarchical search strategy. The subregions of each of the three complexes are densely interconnected by differentially weighted connections.

### 3.5 Principal component analysis

**Figure 9:**
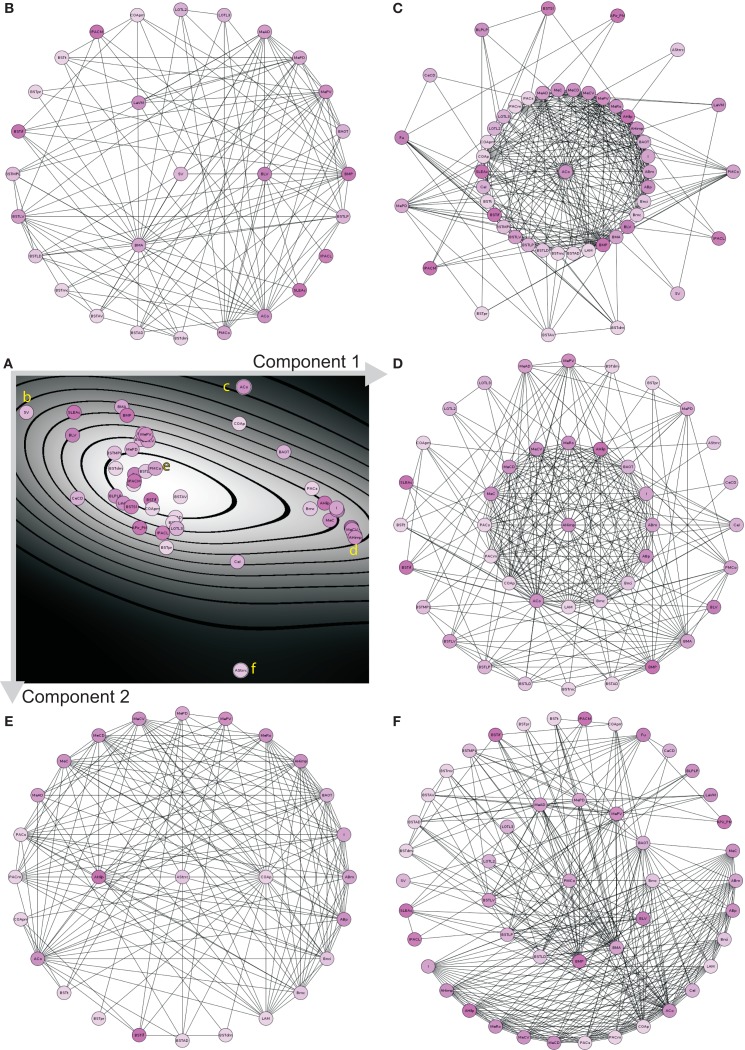
**PCA analysis of the condensed intrinsic amygdala network**. **(A)** Probability density diagram of the 2-dimensional plane of PCA showing the 49 mapped regions. Component axes are indicated and correspond to Table 5. Yellow letters refer to the circle-diagrams. All circle-diagrams have a center circle that correspond to a region of interest of the PCA-plane in **(A)**. The inner-ring of circles are the 1st neighbors and the outer-ring of circles are the 2nd neighbors of the center region. **(B)** Subventricular nucleus (SV). **(C)** Anterior cortical nucleus (ACo). **(D)** Amygdalohippocampal area medial part (AHimp). **(E)** Posteromedial cortical nucleus (PMCo). **(F)** Amygdalostriatal transition area rostrocaudal part.

Principal component analysis (PCA) of the condensed intrinsic amygdala network was applied to detect groups of regions with similar patterns of connectivity (Echtermeyer et al., 2011; Schmitt and Eipert, 2012). The degree all (DG_all_; *all* refers to the sum of input and output connections), average degree of neighbors (AvgDG_nb_; Rubinov and Sporns, 2010), cluster coefficient all (CluC_all_; Rubinov and Sporns, 2010), cluster coefficient of 2nd neighbors (Echtermeyer et al., 2011; CluC_2_), variation coefficient of neighbor degree (VC_DG1_; Echtermeyer et al., 2011), and locality (Loc; da Costa et al., 2006) were used as 6 dimensions (feature vector) for PCA because these local parameters quantify connectivity relations of direct and indirect neighbors. Here, PCA maps feature vectors of all nodes to a plane to detect differences between nodes with regard to their feature expression. A Parzen-window (Echtermeyer et al., 2011) was used to realize a probability density function in the two-dimensional PCA-plane. The contribution of each parameter to the components is shown in Table 5. The parameters locality (Loc), cluster coefficient all (CluC_all_), and average neighbor degree (AvgDG_nb_) are the largest among all 6 variables and have the biggest influence on the x-axis (component 1). The absolute values of parameters degree all (DG_all_) and the variation coefficient of neighbor degree (VC_DG_) have the biggest influence on the y-axis (component 2; Figure 9A). The subventricular nucleus (SV) is located at the upper left corner of the PCA-plane. SV has only three direct neighbors which have a similar amount of connections to 2nd neighbors of SV like the 2nd neighbors among themselves (*1st weak connectivity*). The anterior cortical amygdaloid nucleus (ACo) has a large *degree all* and many connections between 1st neighbors, however, sparse connections from 1st to 2nd neighbors and among 2nd neighbors (*1st NB focused connectivity*). The amygdalohippocampal area medial part (AHimp) has many neighbors which are very densely connected among themselves and more densely than the 2nd neighbors among themselves (1st dense connectivity). A similar connectional pattern can be found for the regions mapped around AHimp: Pacs, Bmc, AHilp, I, MeC, PACm, MeRo, ABm, MeCVABp. The posteromedial cortical nucleus (PMCo) is located almost in the center of the PCA-plane. It has a medium number of connections to direct neighbors and the 2nd neighbors are strongly connected among themselves. In relation to the 1st neighbors, the 2nd neighbors of PMCo are connected more densely (*1st balanced mid connectivity*). Around PMCo is a second larger group of regions that join the latter connectivity feature of PMCo. The bed nuclei of the stria terminalis are among this PMCo-group. The amygdalostriatal transition area rostrocaudal part (AStrrc) is located at the bottom of the PCA-plane and has only two 1st neighbors. The 2nd neighbors of AStrrc are strongly connected among themselves (*2nd NB focused connectivity*).

**Table 5:** **A feature vector consists of the parameters degree all (DG_all_), average degree (AvgDG_nb_), cluster coefficient all (CluC_all_), cluster coefficient of 2nd neighbors (CluC_2_), variation coefficient of neighbor degree (VC_DG_), and locality (Loc; for a definition of parameters, see Echtermeyer et al., 2011)**.

Component	DG_all_	AvgDG_nb_	CluC_all_	CluC_2_	VC_DG_	Loc	Share
1	0.392	0.448	0.457	−0.297	−0.375	0.456	58.893%
2	−0.529	0.266	0.412	0.425	−0.45	−0.312	20.035%
3	0.33	−0.382	−0.107	0.693	−0.324	0.384	13.459%
4	0.164	0.43	0.251	0.483	0.697	0.073	5.918%
5	−0.46	−0.471	0.491	−0.122	0.254	0.496	1.468%
6	0.468	−0.419	0.533	−0.049	0.04	−0.544	0.328%

*For each principal component, the contribution of the feature vector is given for each feature, and by its *Share* (percentage of the variance of a component of the total variance of the 6-dimensional feature space; Echtermeyer et al., 2011)*.

### 3.6 Vulnerability of the intrinsic amygdala network

**Figure 10:**
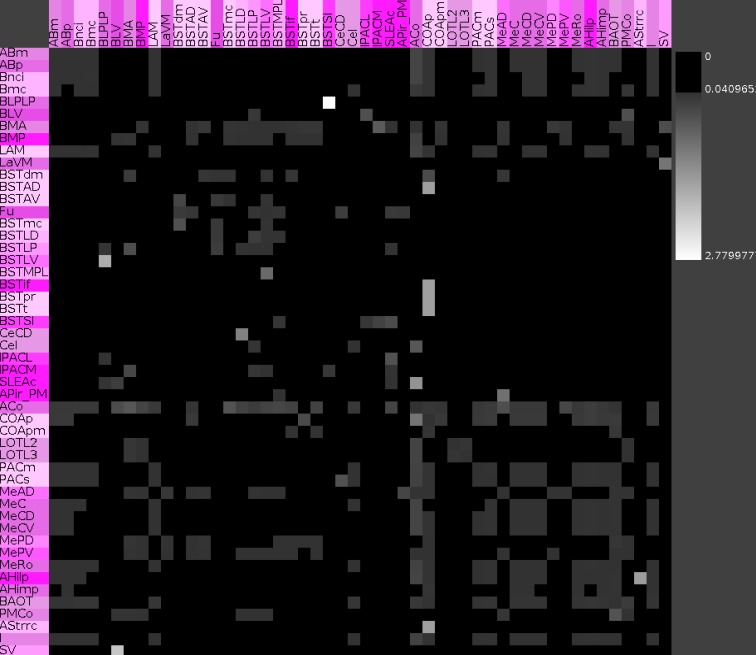
**Vulnerability matrix of the condensed intrinsic amygdala network**. Values indicate the decrease of closeness after removing a particular connection. Removal of the connection from the posterior basolateral nucleus lateral part to the supracapsular bed nucleus of the stria terminalis lateral part has the largest significance of 2.78% (lightest gray value).

Vulnerability analysis in *neuroVIISAS* was applied to identify nodes and connections that contribute significantly to the integrity of the intrinsic amygdala network. The analysis has been performed by iteratively removing nodes (significance list Table 6) or edges (vulnerability matrix Figure 10) from the condensed intrinsic amygdala network, followed by calculating the *closeness* (inverse of the sum of distances to all other nodes) of remaining nodes. The relative change in percent of the closeness turns out to be a meaningful quantity for the significance of removed nodes. If the relative average closeness is large (average path length increases) after removing a node, then this node possesses a large significance for the connectivity of the network. A negative relative average change of closeness (average path length decreases) may occur if the removed node is relatively far away from most other nodes. In Table 6 the posterior amygdaloid nucleus (9.434%), the anterior cortical amygdaloid nucleus (9.227%), and the anterior basomedial nucleus (2.695%) have largest significances and if these nodes are removed the average path length increases much more than removing other nodes. The relative average change of closeness after removing connections is visualized in the vulnerability matrix (Figure 10). The cluster of regions in the lower right quadrant of the vulnerability matrix shows similar values and belongs to the superficial amygdaloid complex.

**Table 6:** **Significance of regions in percent based on the change of closeness after removing a particular region from the network**.

Region	Significance
Posterior amygdaloid nucleus	9.434
Anterior cortical amygdaloid nucleus	9.227
Anterior basomedial nucleus	2.695
Amygdalohippocampal area lateral part	2.325
Bed nucleus of the accessory olfactory tract	1.953
Ventral basolateral nucleus	1.952
Medial amygdaloid nucleus anterodorsal part	1.808
Supracapsular bed nucleus of the stria terminalis lateral part	1.530
Periamygdaloid complex sulcal division	1.422
Central division of sublenticular extended amygdala	1.084
Intercalated nuclei of the amygdala	1.004
Accessory basal nucleus magnocellular division	0.918
Accessory basal nucleus parvicellular division	0.918
Medial amygdaloid nucleus rostral part	0.918
Medial amygdaloid nucleus central dorsal part	0.876
Periamygdaloid complex medial division	0.876
Medial amygdaloid nucleus central ventral part	0.833
Posteromedial cortical nucleus	0.828
Medial amygdaloid nucleus caudal part	0.734
Posterior basomedial nucleus	0.636
Bed nucleus of the stria terminalis lateral division dorsal part	0.616
Lateral nucleus medial part	0.606
Bed nucleus of the stria terminalis lateral division posterior part	0.592
Basal nucleus magnocellular part	0.591
Posterior basolateral nucleus lateral part	0.563
Basal nucleus intermediate division	0.520
Medial amygdaloid nucleus posteroventral part	0.510
Amygdalohippocampal area medial part	0.289
Bed nucleus of the stria terminalis anterior division dorsomedial nucleus	0.254
Bed nucleus of the stria terminalis lateral division ventral part	0.163
Bed nucleus of the stria terminalis fusiform part	0.020
Medial amygdaloid nucleus posterodorsal part	−0.073
Central amygdaloid nucleus intermediate division	−0.098
Bed nucleus of the stria terminalis magnocellular nucleus	−0.280
Bed nucleus of the stria terminalis anterior dorsal area	−0.304
Posterior amygdaloid nucleus medial part	−0.326
Interstitial nucleus of the posterior limb of the anterior commissure medial part	−0.399
Subventricular nucleus	−0.448
Bed nucleus of the stria terminalis interfascicular nucleus	−0.454
Bed nucleus of the stria terminalis transverse nucleus	−0.539
Bed nucleus of the stria terminalis principal nucleus	−0.603
Bed nucleus of the stria terminalis anterior ventral area	−0.634
Amygdalopiriform transition area posteromedial part	−0.949
Bed nucleus of the stria terminalis medial division posterolateral part	−1.013
Amygdalostriatal transition area rostrocaudal part	−1.050
Interstitial nucleus of the posterior limb of the anterior commissure lateral part	−1.170
Central amygdaloid nucleus caudal division	−1.270
Ventromedial part of the lateral nucleus	−1.623
Nucleus of the lateral olfactory tract layer 2	−1.777
Nucleus of the lateral olfactory tract layer 3	−1.777

### 3.7 Extrinsic connectivity of the amygdala

The condensed intrinsic network of an unilateral amygdala has ipsilateral and contralateral inputs and outputs to regions that do not belong to the intrinsic amygdala connectome. *Direct* connections start at leafs (regions that are not split into subregions or where subregions are not opened in the user-defined hierarchy presentation) of subtrees and were considered to calculate extrinsic inputs and outputs. The anterior cortical amygdaloid nucleus has the largest direct ipsilateral input (101) and the second largest direct contralateral input (11). The direct ipsilateral output consists of 138 and the direct contralateral output consists of 19 connections (rank 5). The basal nucleus intermediate division has the most directed contralateral outputs (37), yet, only 19 ipsilateral outputs (rank 29). The anterior cortical amygdaloid nucleus turns out to be a part of the condensed intrinsic amygdala network that has most ipsilateral inputs and outputs. Table 7 documents that ipsilateral and contralateral connectivity is not positively correlated for each region, e.g., the interstitial nucleus of the posterior limb of the anterior commissure medial part.

**Table 7:** **Extrinsic bilateral inputs and outputs of all regions of the condensed intrinsic amygdala network**.

	Dic	Dii	Dis	Doc	Doi	Dos	Sic	Sii	Sis	Soc	Soi	Sos
ACo	11	101	112	19	138	157	11	101	112	19	138	157
COAp	5	70	75	15	71	86	8	73	81	15	71	86
IPACM	14	60	74	1	64	65	14	60	74	1	64	65
PMCo	4	51	55	7	47	54	4	51	55	7	47	54
BMP	4	47	51	23	113	136	4	61	65	23	113	136
BLV	4	42	46	9	39	48	4	42	46	9	39	48
BSTLV	3	42	45	1	28	29	3	42	45	1	28	29
BMA	1	38	39	10	107	117	1	43	44	10	107	117
I	3	33	36	0	16	16	4	33	37	0	16	16
LaVM	4	30	34	4	23	27	4	30	34	4	23	27
MeAD	4	30	34	10	66	76	4	30	34	10	66	76
BSTAD	7	22	29	0	0	0	7	22	29	0	0	0
SLEAc	2	27	29	0	20	20	2	27	29	0	20	20
LAM	2	26	28	1	43	44	2	26	28	1	43	44
BSTLD	6	21	27	1	37	38	6	21	27	1	37	38
BSTLP	3	23	26	1	19	20	3	23	26	1	19	20
CeI	2	24	26	3	18	21	2	24	26	3	18	21
BSTt	8	15	23	0	0	0	8	17	25	0	0	0
BSTif	8	14	22	0	1	1	8	16	24	0	1	1
Bmc	2	17	19	6	53	59	8	24	32	6	53	59
MePV	0	19	19	11	63	74	0	19	19	11	63	74
MePD	1	14	15	4	37	41	1	14	15	4	37	41
Bnci	3	11	14	37	19	56	3	11	14	37	19	56
BAOT	1	13	14	0	16	16	1	22	23	0	16	16
ABp	0	14	14	2	44	46	0	14	14	2	44	46
BSTpr	5	8	13	0	2	2	5	8	13	0	2	2
MeC	1	12	13	0	10	10	1	12	13	0	10	10
MeCD	4	8	12	0	10	10	4	8	12	0	10	10
PACs	3	9	12	0	30	30	3	9	12	0	30	30
ABm	2	10	12	10	42	52	2	10	12	10	42	52
AHilp	2	10	12	0	23	23	2	10	12	0	23	23
MeRo	1	10	11	0	8	8	1	10	11	0	8	8
AHimp	0	11	11	2	16	18	0	11	11	2	16	18
BSTAV	5	5	10	0	27	27	5	5	10	0	27	27
PACm	3	7	10	4	30	34	3	7	10	4	30	34
BSTMPL	0	10	10	0	3	3	0	10	10	0	3	3
BSTSl	0	10	10	0	17	17	0	10	10	0	17	17
MeCV	2	7	9	0	11	11	2	7	9	0	11	11
SV	4	4	8	0	0	0	4	4	8	0	0	0
LOTL2	3	4	7	26	36	62	3	4	7	26	36	62
BSTdm	1	6	7	0	71	71	1	6	7	0	71	71
BSTmc	1	6	7	0	44	44	1	6	7	0	44	44
Fu	0	6	6	31	84	115	0	6	6	31	84	115
BLPLP	0	4	4	0	2	2	0	4	4	0	2	2
CeCD	0	4	4	0	6	6	0	4	4	0	6	6
LOTL3	1	2	3	18	39	57	1	2	3	18	39	57
IPACL	1	1	2	0	21	21	1	1	2	0	21	21
APir_PM	0	1	1	0	15	15	0	1	1	0	15	15
COApm	0	1	1	4	18	22	0	1	1	4	18	22
AStrrc	0	0	0	0	0	0	0	0	0	0	0	0

*The extrinsic connections are sorted by the sum of direct inputs. Dic, direct input from contralateral hemisphere; Dii, direct input from ipsilateral hemisphere without regions of the intrinsic amygdala network; Dis, direct input of ipsilateral and contralateral regions; Doc, direct output from contralateral hemisphere; Doi, direct output from ipsilateral hemisphere without regions of the intrinsic amygdala network; Dos, direct output of ipsilateral and contralateral regions; *Sic*, direct input from subtrees of contralateral hemisphere; Sii, direct input from subtrees of ipsilateral hemisphere without regions of the intrinsic amygdala network; Sis, direct input of subtrees of ipsilateral and contralateral regions; Soc, direct output from subtrees of contralateral hemisphere; Soi, direct output from subtrees of ipsilateral hemisphere without regions of the intrinsic amygdala network; Sos, direct output from subtrees of ipsilateral and contralateral regions*.

## 4 Discussion

Analysis and visualization of neuronal connections with regard to networks and connectomes have been pushed forward in the last 30 years (MacDonald, 1983; Felleman and Essen, 1991; Tononi et al., 1994; Young et al., 1994). Specific connectivity analysis based on meta-studies have been performed on the nucleus of the solitary tract of the rat (Palombi et al., 2006), hippocampus of the rat (Burns and Young, 2000), thalamocortical connectivity (Scannell et al., 1999; da Costa et al., 2006), brainstem reticular formation (Humphries et al., 2006), and retrosplenial cortex (Sugar et al., 2011).

In an ongoing meta-study of tract-tracing publications of the rat nervous system, the connectivity data of 2100 articles are collected in *neuroVIISAS*, a generic platform for digital atlasing, connectivity analysis and visualization (Schmitt et al., 2012), and population based simulations. In all tract-tracing studies of the amygdala, groups of regions were described as subdivisions and parts of regions. Therefore, regions of the nervous system are arranged in a neuroontology within *neuroVIISAS*. Because most tract-tracing studies have been performed in the rat, these rat-specific articles have been evaluated.

The total number of peer-reviewed publications of tract-tracing studies of the rat, without considering other organisms, can be determined by convenient expressions that filter the PubMed database (see text footnote 1). 4528 Of such articles can be found in September 2012. 491 Articles of these 4528 mention amyg* (amygdaloideum, amygdaloid, etc.) in their abstracts. Hence, they can be considered as the core of the meta-analysis of tract-tracing based connections of the amygdala. In addition, the bibliographies of review articles and monographs (de Olmos et al., 2004) have been evaluated. In other projects, the connectomes of further functional systems (basal ganglia, cerebellum, cerebral cortex, hippocampus, spinal cord, peripheral nervous system) are analyzed. In many of these tract-tracing publications that do not use the term amyg* in their abstract, input and output connections to and from the amygdala are described and have been captured in the whole rat connectome project. Interestingly, the rat is the organism in which most peer-reviewed tract-tracing studies are available.

Comparable connectome studies of the rat are considering neurons (Arenas et al., 2008) or several areas (whole brain and/or whole CNS; Bohland et al., 2009). The painstaking work of connectivity evaluation of Burns (1997) is based on the terminology of Swanson and Petrovich (1998), ranging at a comparable granularity as the terminology of Paxinos and Watson (2007). The systematic evaluation and databasing of connectivities of the rat brain was proposed and extensively investigated by Burns (1997). This work has been refined by Burns and Cheng (2006), Burns et al. (2008), and Bota and Swanson (2008). So far, a connectome analysis based on a meta-study of tract-tracing publications of the rat amygdala has not been performed.

A principal difficulty of extracting *source-target-weight data* from tract-tracing publications is the definition of interlinked source and target regions in different studies. This is aggravated by the fact that ambiguities of region definitions may occur in different contexts:

1. spatial ambiguities
2. meaning and definition of region terms (ambiguity of definition)
3. location of a region term in a hierarchical terminology (ambiguity of classification)
4. comparative or interspecies alignment of terms (e.g., application of Brodmann area terms of human cerebral cortex parcelation to the rat cerebral cortex; ambiguity of homology)

These particular terminological contexts are the cause for the *correspondence* or *concordance problem* (Stephan et al., 2000; Bezgin et al., 2009; Bohland et al., 2009). An adaptive solution of the problem with regard to the development of neuroanatomical knowledge of regions of nervous systems of different species is not available. The objective relation transformation (ORT; Stephan et al., 2000) reduces the pattern of spatial overlap between region pairs to a discrete set of relational attributes containing identity, subset, superset, and partial overlapping. To realize ORT, a documentation of spatial features (e.g., extension, neighboring regions, super regions, subregions, development) of regions in tract-tracing publications is necessary. Unfortunately, the objective of tract-tracing studies is a description of sources, targets, and pathways visualized by axonal transport of tracing substances. Hence, a definition of regions and the comparison of them with other studies is out of scope in most cases (Pitkänen, 2000; Jones et al., 2005). Data that are necessary to apply ORT consistently are either missing in most tract-tracing studies or they must be inferred (indirect availability). The latter may also contribute to imponderables and variability in region definitions.

In this work, extracted *source-target-weight data* are directly derived from the original text to reduce inconsistencies as much as possible. This has the advantage of reproducibility of sources and targets in a connectome. Interpretations of how much a region partially overlaps with regions in other publications or if subregions are assigned to different superregions are avoided to prevent errors introduced by misinterpretations. Furthermore, necessary data that allow correct interpretation of terminological overlap and subdivision problems are often missing in publications. Hence, source, target, and weight data are extracted with a minimum of further interpretation to accumulate them continuously during evaluation of new tract-tracing publications. By using accumulated data, it is possible to generate different selections of nodes from the whole neuroontology of the rat nervous system. In *neuroVIISAS*, several mechanisms to assemble regions of the complete neuroontology have been implemented to generate problem-dependent adjacency matrices. A coarse selection of superregions (e.g., thalamus, hypothalamus, sensomotoric cortex, cerebellum) for connectivity analysis provides more validity with regard to region definitions (comparable with tract-tracing data judgment in ORT), however, less important and relevant connectivity information is the result. Nevertheless, the approach of accumulating raw data from original publications in a consistent framework avoids the introduction of errors while solving overlap and classification problems if necessary data are not available for the problem at hand.

Beside the concordance problem, there are further factors that constrain the informative value of tract-tracing based connectome construction:

1. different dynamics of tracer transport
2. selectivity of tracer transport (unidirectional, bidirectional)
3. identification of axonal terminals and fibers of passages
4. tracer uptake by damaged fibers of passage
5. technical details of tracer application (volume, velocity, iontophoresis)
6. distance of sources and targets from the location of tracer application and survival time
7. spread of tracer in adjacent regions (especially in stereotaxic pressure injections)
8. sensitivity of tracer and immunohistochemical visualization
9. semiquantitative and qualitative interpretation of connection strength (weight) is not normalized between different studies and most criteria for weights are not based on stereological and densitometric evaluations
10. investigator bias (interpretation of descriptive representations of tract-tracing results)

The amygdala connectome has been analyzed using *neuroVIISAS* by several global and network measures, matrices, motifs, and multivariate techniques. However, other methods are available to investigate the structures and features of complex networks (Kötter and Stephan, 2003; Tononi and Sporns, 2003; Sporns and Kötter, 2004; Sporns and Zwi, 2004; Bassett and Bullmore, 2006; Nagyessy et al., 2006; da Costa et al., 2007; Kötter et al., 2007; Sporns et al., 2007). More specifically, neural complexity (Tononi et al., 1994), community structure (Newman and Girvan, 2004), connectivity descriptors (Kammer and Täubig, 2004; Goryczka and Arodz, 2006), functional topology analysis (Blinder et al., 2005), efficiency and cost of networks (Achard and Bullmore, 2007), node conformity of factorizable networks (Dong and Horvath, 2007), lesion analysis, and transfer entropy (Schreiber, 2000; Honey and Sporns, 2008) as well as spectral analysis of networks (Baltz and Kliemann, 2004) are advanced methods to reveal structures and functions of networks. In addition, multivariate methods of non-metric multidimensional scaling and Procrustes *R*^2^ statistics (Goodhill et al., 1995; Burns and Young, 2000), hierarchical optimization (Hilgetag et al., 1996; Burns and Young, 2000), optimal hierarchical orderings (Hilgetag et al., 2000), and canonical variable analysis (da Costa et al., 2007) are options to further investigate results that are obtained by the principal component analysis as found here.

By selecting those regions where inputs and outputs within the intrinsic amygdala network are reported, a condensed intrinsic amygdala network was generated. This network is a scale-free and, to some degree, small-world network. The adjacency matrix and complexer matrices exhibit a cluster of connections (Figure 5) within the subregions of the superficial amygdaloid cortex which can also be observed at different levels of the region hierarchy (Figure 4). The medial amygdaloid nucleus has the largest number of efferents and afferents within this cluster, described here for the first time. The dense intraamygdaloid connection of the medial amygdaloid nucleus and the central amygdaloid nucleus that have also been reported in the review of Pitkänen (2000) can be confirmed here (subsection *4.2. Network*, Figure 5). These densely interlinked regions of the superficial amygdaloid cortex belong to olfaction, autonomic hypothalamus and prefrontal cortex cognition.

The CE, ME, BL, and LA regions are considered as hodologic and functional entities of the amygdala (Aggleton, 2000; Pitkänen, 2000). Based on the subdivision of de Olmos et al. (2004), the resulting connectome was modified to obtain CE, ME, BL, and LA regions as connectional nodes. CE and ME have comparable quantitative features with regard to local parameters (indegrees, outdegrees, Shapley rates) and cycle counts. This indicates that these regions are very important with regard to integrity of the intrinsic amygdala connectome. In addition, their frequency of participation for the complete reciprocal motif 3-13 is larger in comparison to other regions. In contrast, BL and LA have lower Shapley rates and they do not participate as often as CE and ME to 2 cycle and 3 cycle pathways within the intrinsic connectome of the amygdala. However, BL receives the second most extrinsic inputs from non-amygdala regions of the ipsi- and contralateral hemisphere. From the connectional perspective this corresponds with the point of view that BL and LA are *entries* of sensory information (Kim and Jung, 2006). Furthermore, BL participates most often in the 3-04 motif with one reciprocal and a single non-reciprocal edge. The reciprocal edge can be interpreted in terms of a local regulatory function (positive or negative feedback) and the connection to a node that does not project to the reciprocally connected nodes could be regarded as a regulatory output of this motif. To summarize a connectional role of BL, we can consider it as an extrinsic receiver that may regulate extrinsic inputs and then distribute them in the intrinsic connectome of the amygdala. ME possesses second most extrinsic outputs and a relatively small Shapley rate which could emphasize the connectional role as a sender to extrinsic targets and a strong intrinsic network entity. However, CE receives and sends most extrinsic inputs and outputs and has the smallest Shapley rate which points to a more integrative function in comparison to BL and ME. These interpretations are speculative and need to be verified. Nevertheless, they are based on a huge number of observations in tract-tracing studies.

The sensory inputs from the somatosensory, visual, and auditory cortex to the LA have been confirmed in the connectome data. Furthermore, we can agree with dense interconnections between LA and basal complex regions. Reciprocal connections between LA and Bnc (BL is a subregion of Bnc) were also found. Dense interconnections with reciprocal projections between Bnc and CE are found as well. Hence, the pathway of fear conditioning (Kim and Jung, 2006; Ciocchi et al., 2010; Haubensak et al., 2010; Tye and Deisseroth, 2012) from different sensory regions can be reconstructed. However, it turns out that there are a lot more interconnections between specific subregions and reciprocal projections are known than considered in Haubensak et al. (2010), Ciocchi et al. (2010), Tye and Deisseroth (2012), and Kim and Jung (2006).

The Shapley rate is an indicator for the importance of a particular region in the network. Regions which posses largest importance with regard to the Shapley rate are the posterior amygdaloid nucleus, the anterior cortical amygdaloid nucleus, and the supracapsular bed nucleus of the stria terminalis lateral part. However, the latter has only a small *degree all* of 13 connections. Hence, Shapley rates should always be judged in comparison with other local parameters. Also, the Shapley rates of the bilateral condensed intrinsic amygdala network indicate that these regions are as important as those in the unilateral network.

The complete reciprocal 3-node and 4-node motifs occur significantly more often in the real condensed amygdala network than in rewiring simulations, a finding reported here for the first time. However, circular motifs without reciprocal connections are less abundant in the real network. Nodes that possess large centrality values and importance for the network structure are always involved in the completely reciprocal motifs. This could indicate a strong regulatory role and self control by connectional dense feedbacks.

By removing the posterior amygdaloid nucleus, the anterior cortical amygdaloid nucleus, and the anterior basomedial nucleus, strongest changes of relative closeness have been observed. However, the anterior basomedial nucleus does not have such strong centrality features like the regions with lowest Shapley rates, indicating that vulnerability analysis may provide additional information regarding the role of regions that has not been revealed by quantities of local network analysis.

In the PCA we found that the anterior cortical amygdaloid nucleus has many 1st neighbors, however, connectivity between 1st and 2nd neighbors is weak. In contrast, the amygdalohippocampal area medial part has many 1st neighbors and they are also strongly interconnected. The posteromedial cortical amygdaloid nucleus has numerous 1st neighbors which are strongly interconnected. In addition, the 2nd neighbors of PMCo are densely interconnected, too. These features of local connectivity of PMCo in relation to its smallest Shapley rate of the intrinsic amygdala connectome indicate its important role in the network.

Finally, it should be emphasized that the connectional information accumulated from the evaluation of a large amount of tract-tracing publications may only reflect partial trends in connectivity detection of the tract-tracing community. The real and complete connectome of the rat amygdala might have features that cannot be detected by analysis of all published tract-tracing data. However, by also including publications in this work which are not focused on tracer injections in the amygdala, validity of the basic network structure is expected to become larger. Further limitations of neural connectome analysis are the spatiotemporal dynamics of connectivity at the synaptic level.

In conclusion, the quantitative analysis of the amygdala connectome allows to identify regions of the superficial amygdaloid complex that are densely interlinked and may be important for the internal regulation of information processing of the intrinsic amygdala network. The amygdala network has small-word and scale-free properties. It contains complete reciprocal motifs significantly more frequently than in randomizations. The posterior amygdaloid nucleus, anterior cortical amygdaloid nucleus, and bed nucleus of the accessory olfactory tract turned out to send and receive most connections within the condensed amygdaloid network.

## Conflict of Interest Statement

The authors declare that the research was conducted in the absence of any commercial or financial relationships that could be construed as a potential conflict of interest.

## Footnotes

^1^http://www.ncbi.nlm.nih.gov/pubmed

^2^http://jabref.sourceforge.net/

## A Appendix

The definitions of expressions, parameters, matrices, and simulation models (random graph models) used in the article are summarized in the following.

### A.1 Basic definitions

#### A.1.1 Node, vertex

The smallest subunit of a network. With regard to connectomes a node is a circumscribed or disjunctive region that contains neuron perikarya (sources of physiological action potential) and/or axonal terminals (targets of physiological action potentials).

#### A.1.2 Set of indexed nodes

The set of all indices of nodes is

(A1) $$N=\left\{\text{1,}\;\text{2,}\;\text{3,}\;...\text{,}\;n\right\}$$

#### A.1.3 Number of nodes

The number of nodes (regions, vertices) is

(A2) n=|N|

#### A.1.4 Edge

A directed edge (*i*, *j*) ∈ *N* × *N* is the line that connects vertices *i* and *j* with source *i* and target *j*. The set of directed edges *E* is

(A3) E⊆N×N

#### A.1.5 Edges

The number of edges (connections, links) ϵ is

(A4) ϵ=|E|

#### A.1.6 Set of edges

The set of all not self-referencing edges is

$$\begin{array}{rcll} & L=\left\{\left(i,j\right)\in E\left|i\neq j\right.\right\} & & \text{(A5)}\\ & \ell =|L| & & \text{(A6)} \end{array}$$

#### A.1.7 Graph

(A7) $$G=\left(N,\;E\right)$$

#### A.1.8 Adjacency matrix

The adjacency matrix (connectivity matrix) A is

(A8) $$A={\left(a_{ij}\right)}_{i,j=1}^{n}\;\;\text{where}\;a_{ij}=\left\{\begin{array}{cc} 1\; & \text{if}\;\left(i,\;j\right)\in E\\ 0\; & \text{else}\\ \; \end{array}\right.$$

#### A.1.9 Weighted matrix

The weighted matrix W is

(A9) $$W={\left(w_{ij}\right)}_{i,j=1}^{n}$$

whereby *w_ij_* is the weight of the edge (*i*, *j*) that connects *i* and *j*. 0 ≤ *w_ij_* ≤ 1.

#### A.1.10 Path

A sequence of vertices (*v*_1_, …, *v_k_*) is a path from (*v*_1_ to *v_k_*) if ∀*_i_* ∈ {1, …, *k* − 1}: (*v*_1_, *v*_*i*+1_) ∈ *E*. The length of a path *v*_1_, …, *v_k_* is *k* − 1.

#### A.1.11 Distance matrix

The distance matrix D is

(A10) $$D={\left(d_{ij}\right)}_{i,j=1}^{n}$$

where

(A11) $$d_{ij}=d\left(i,j\right)=\left\{\begin{array}{cc} \text{length of the shortest}\;\\ \;\text{path from }i\text{ to }j,\; & \text{if such a path exists}\\ \infty ,\; & \text{else}\\ \; \end{array}\right.$$

#### A.1.12 Degree all

Self-references of nodes are not considered for all three degree measures. deg(*i*) = *deg_all_*(*i*)

(A12) $$\deg\left(i\right)=\overset{n}{\underset{{}_{j\neq i}^{i=1}}{\sum }}a_{ij}+a_{ji}$$

#### A.1.13 Degree out

(A13) $$\deg_{out}\left(i\right)=\overset{n}{\underset{\begin{array}{c} i=1\\ j\neq i \end{array}}{\sum }}\;a_{ij}$$

#### A.1.14 Degree in

(A14) $$\deg_{in}\left(i\right)=\overset{n}{\underset{\begin{array}{c} i=1\\ j\neq i \end{array}}{\sum }}\;a_{ji}$$

#### A.1.15 Neighborhoods

Out-neighbors of *i*:

(A15) $$N_{i}^{out}=\left\{j\in N\backslash \left\{i\right\}\;|\;a_{ij}=1\right\}$$

In-neighbors of *i*:

(A16) $$N_{i}^{in}=\left\{j\in N\backslash \left\{i\right\}\;|\;a_{ji}=1\right\}$$

All neighbors of *i*:

$$\begin{array}{rcll} & N_{i}=N_{i}^{out}\cup N_{i}^{in} & & \text{(A17)}\\ & N_{i}^{+}=N_{i}\cup \left\{i\right\} & & \text{(A18)} \end{array}$$

### A.2 Network parameters

#### A.2.1 Leverage Lev(i)

The leverage centrality was introduced by Joyce et al. (2010).

(A19) $$Lev\left(i\right)=\frac{1}{deg\left(i\right)}\underset{j\in N_{i}}{\sum }\frac{deg\left(i\right)-deg\left(j\right)}{deg\left(i\right)+deg\left(j\right)}$$

#### A.2.2 Directed leverage $Lev{\left(i\right)}^{\to }$

(A20) $$Lev{\left(i\right)}^{\to }=\frac{1}{\left|N_{i}\right|}\underset{j\in N_{i}}{\sum }\frac{deg_{all}\left(i\right)-deg_{all}\left(j\right)}{deg_{all}\left(i\right)+deg_{all}\left(j\right)}$$

The weighted directed leverage $Lev{\left(i\right)}^{\vec{w}}$ is analogue to $Lev{\left(i\right)}^{\to }$ with weighted degrees.

#### A.2.3 Communicability matrix *G*

(A21) $$G_{ij}=\overset{\infty }{\underset{k=0}{\sum }}\frac{{\left(A^{k}\right)}_{ij}}{k!}={\left(e^{A}\right)}_{ij}$$

The communicability is the sum of paths from *i* to *j* each weighted with $\frac{1}{k!}$ for a path with length *k*.

#### A.2.4 Modularity measure

Let *M* = {*M*_1_, …, *M_m_*} be a partition of *N*. *M_i_* is a group, module, or cluster of vertices. With

(A22) $$e_{i}=\frac{1}{\ell }\underset{\begin{array}{c} j,k\in M_{i}\\ j<k \end{array}}{\sum }\left(a_{jk}+a_{kj}\right),$$

the fraction of edges that fall within group *M_i_* ⊆ *N* and

(A23) $$a_{i}=\frac{1}{2\ell }\underset{j\in M}{\sum }\;\underset{k\in N\backslash \left\{j\right\}}{\sum }\;\left(a_{jk}+a_{kj}\right),$$

the fraction of ends of edges that are attached to vertices in group *M_i_*, the modularity

(A24) $$Q=\overset{m}{\underset{i=1}{\sum }}\left(e_{i}-a_{i}^{2}\right),$$

whereby $a_{i}^{2}$ is the fraction of edges that would connect vertices within group *M_i_* if they were connected at random. A large modularity implies that the fraction of edges that fall within groups is larger than expected in the random case. The partition is generated by a “greedy” optimization algorithm. Starting with a partition where every single node has its own group, stepwise those two groups are joined that increase *Q* most. The algorithm ends if there are no more such groups. The weighted case is similar, only the *a_ij_* are replaced by *w_ij_* and ℓ is replaced by the sum of the edge weights

(A25) $$\ell^{\vec{w}}=\underset{\begin{array}{c} i,j\in N\\ i\neq j \end{array}}{\sum }w_{ij}$$

The method of Newman and Girvan (2004) was used.

#### A.2.5 Global efficiency (*GE*)

(A26) $$GE=\frac{1}{n\left(n-1\right)}\cdot \underset{\begin{array}{c} i,j\in N\\ i\neq j \end{array}}{\sum }\frac{1}{d\left(i,\;j\right)}$$

*GE*^→^ and $GE^{\vec{w}}$ analog with *d*^→^(*i*, *j*) and $d^{\vec{w}}\left(i,j\right)$

#### A.2.6 Directed global efficiency

(A27) $$GE^{\to }=\frac{1}{n\left(n-1\right)}\cdot \underset{\begin{array}{c} i,j\in N\\ i\neq j \end{array}}{\sum }\frac{1}{d^{\to }\left(i,\;j\right)}$$

#### A.2.7 Harmonic mean (*HM*)

(A28) $$HM=\frac{1}{GE}$$

The directed and weighted versions use the directed and weighted global efficiencies.

#### A.2.8 Local efficiency

The local efficiency indicates how strong neighbors of nodes are interconnected. For each node *i* the inverse lengths of the shortest paths of the neighbors of *i* that are passing *i* are added. The local efficiency is this sum divided by the maximal possible sum of paths between neighbors that are connected with *i*. The local efficiency of the network is the average local efficiency of all nodes.

#### A.2.9 Directed local efficiency

(A29) $$LE^{\to }=\frac{1}{n}\underset{\begin{array}{c} i\in N\\ n_{i}>1 \end{array}}{\sum }\frac{\underset{\begin{array}{c} j,k\in N_{i}\\ j\neq k \end{array}}{\sum }d_{jk}{\left(N_{i}\right)}^{-1}}{n_{i}\cdot \left(n_{i}-1\right)}$$

#### A.2.10 Weighted directed local efficiency

(A30) $$LE^{\vec{w}}=\frac{1}{n}\underset{\begin{array}{c} i\in N\\ n_{i}>1 \end{array}}{\sum }\frac{\underset{\begin{array}{c} j,k\in N_{i}\\ j\neq k \end{array}}{\sum }d_{jk}^{\vec{w}}{\left(N_{i}\right)}^{-1}}{n_{i}\cdot \left(n_{i}-1\right)}$$

Whereby *n_i_* = |*N_i_*| and *d_jk_*(*N_i_*), respectively, $d_{jk}^{\vec{w}}\left(N_{i}\right)$ is the length of the shortest path between *j* and *k* that contains only neighbors of *i*.

#### A.2.11 Directed assortativity coefficient ${\text{r}}^{\;\to }$

(A31) $$r^{\to }=\frac{\begin{array}{c} \underset{\left(i,\;j\right)\in L}{\sum }\deg_{out}\left(i\right)\cdot \deg_{in}\left(\;j\right)\\ \;-\frac{1}{4\ell }\cdot {\left[\underset{\left(i,\;j\right)\in L}{\sum }\left(\deg_{out}\left(i\right)+\deg_{in}\left(\;j\right)\right)\right]}^{2} \end{array}}{\begin{array}{c} \frac{1}{2}\cdot \underset{\left(i,\;j\right)\in L}{\sum }\left(\deg_{out}{\left(i\right)}^{2}+\deg_{in}{\left(\;j\right)}^{2}\right)\\ \;-\frac{1}{4\ell }\cdot {\left[\underset{\left(i,\;j\right)\in L}{\sum }\left(\deg_{out}\left(i\right)+\deg_{in}\left(\;j\right)\right)\right]}^{2} \end{array}}$$

#### A.2.12 Directed and weighted assortativity coefficient $r^{\vec{w}}$

(A32) $$r^{\vec{w}}=\frac{\begin{array}{c} \underset{\left(i,j\right)\in L}{\sum }\;w_{ij}\cdot \left(\deg_{out}^{w}\left(i\right)\cdot \deg_{in}^{w}\left(\;j\right)\right)\\ \;-\frac{1}{4\ell }\cdot {\left[\underset{(\mathrm{i,j})\in L}{\sum }\;w_{\mathrm{ij}}\cdot \left(\deg_{\mathrm{out}}^{w}(i)+\deg_{\mathrm{in}}^{w}(\;j)\right)\right]}^{2} \end{array}}{\begin{array}{c} \frac{1}{2}\cdot \underset{\left(i,j\right)\in L}{\sum }\;w_{ij}\cdot \left(\deg_{out}^{w}{\left(i\right)}^{2}+\deg_{in}^{w}{\left(\;j\right)}^{2}\right)\\ \;-\frac{1}{4\ell }\cdot {\left[\underset{(\mathrm{i,j})\in L}{\sum }\;w_{\mathrm{ij}}\cdot \left(\deg_{\mathrm{out}}^{w}(i)+\deg_{\mathrm{in}}^{w}(\;j)\right)\right]}^{2} \end{array}}$$

The correlation of the degrees of nodes that are connected: −1 ≤ *r* ≤ 1. Large positive values imply that nodes are mainly connected to nodes with similar degrees. Large negative values imply that nodes with a large degree are mainly to nodes that have a small degree. If *r* ≈ 0 there is no relation detectable.

#### A.2.13 Average path length = characteristic path length $\bar{d}$

With *P* = {(*i*, *j*) ∈ *N* × *N* | *d*(*i*, *j*) < ∞}, the set of paths.

(A33) $$\bar{d}=\left\{\begin{array}{cc} \frac{1}{\left|P\right|}\underset{\left(i,\;j\right)\in P}{\sum }d\left(i,\;j\right),\; & P\neq \emptyset \\ 0,\; & P=\emptyset \\ \; \end{array}\right.$$

In the weighted case the distances *d(i,j)* are replaced by the weighted distances.

#### A.2.14 Average directed degree (*deg*)

(A34) $$\bar{deg}=\frac{2\ell }{n}$$

#### A.2.15 Heterogeneity *VC*

Coefficient of variation (*VC*) of the *degree_all_* parameter.

(A35) $$H_{VC}=\frac{1}{\bar{deg}}\cdot \sqrt{{\underset{i\in N}{\sum }\left(deg_{all}\left(i\right)-\bar{deg}\right)}^{2}}$$

If *H_VC_* = 0, all nodes have the same degree. The larger *H_VC_* the more diverse are the node degrees. In the weighted case the versions of the degrees are used. The heterogeneity measure of Estrada (2010) was not implemented because it is not defined for directed and weighted graphs.

#### A.2.16 Line density (*Ld*)

The line density is the same like the connectedness of Raghuraj and Lakshminarayanan (2006).

(A36) $$Ld=\frac{\ell }{n\cdot \left(n-1\right)}$$

Without self-referencing edges.

#### A.2.17 Diameter (*Diam*)

(A37) $$Diam=\max\left\{d\left(i,\;j\right)|d\left(i,\;j\right)<\infty \right\}$$

#### A.2.18 Katz index

The Katz index (Katz status index, Katz centrality) is a measure for the direct and indirect input of a node.

(A38) $$C_{Katz}\left(i\right)=\overset{\infty }{\underset{k=1}{\sum }}\underset{j=1}{\sum }\alpha^{k}{\left(A^{k}\right)}_{ji}$$

The attenuation factor α has to be smaller than the reciprocal of the absolute value of the largest eigenvalue of *A*. For a better readability and comparability of the results, in neuroVIISAS the Katz centrality is multiplied by the mean of the quotient deg*_in_*(*i*)/*C_Katz_*(*i*) of all nodes with *C_Katz_*(*i*) > 0. Hence, the values lie in the same range as the indegrees.

#### A.2.19 Number of triangles

(A39) $$t^{\to }\left(i\right)=\underset{\begin{array}{c} j,k\in N\backslash \left\{i\right\}\\ j<k \end{array}}{\sum }\left(a_{ij}+a_{ji}\right)\left(a_{ik}+a_{ki}\right)\left(a_{jk}+a_{kj}\right)$$

The maximum number of possible triangles that can be derived from a complete reciprocal triangle is 8.

#### A.2.20 Weighted number of triangles

(A40) $$t^{\vec{w}}\left(i\right)=\underset{\begin{array}{c} j,k\in N\backslash \left\{i\right\}\\ j<k \end{array}}{\sum }\left(w_{ij}^{\frac{1}{3}}+w_{ji}^{\frac{1}{3}}\right)\left(w_{ik}^{\frac{1}{3}}+w_{ki}^{\frac{1}{3}}\right)\left(w_{jk}^{\frac{1}{3}}+w_{kj}^{\frac{1}{3}}\right)$$

Instead of the sum of triangles [*t*^→^(*i*)] the sum of geometric means of edge weights of each triangle is calculated. The following example provides (*w_ij_* · *w_jk_* · *w_ik_*)^1/3^ as the summand:

#### A.2.21 Directed transitivity

The general definition of transitivity (*T*) is the sum of number of triangles around all nodes divided by the maximum possible sum of triangles around all nodes.

(A41) $$T^{\to }=\frac{\underset{i\in N}{\sum }t^{\to }\left(i\right)}{\underset{i\in N}{\sum }t_{\max}\left(i\right)}$$

#### A.2.22 Directed and weighted transitivity

(A42) $$T^{\vec{w}}=\frac{\underset{i\in N}{\sum }t^{\vec{w}}\left(i\right)}{\underset{i\in N}{\sum }t_{\max}\left(i\right)}$$

Whereby *t*_max_(*i*) =  deg(*i*)·(deg(*i*) − 1) − 2·rec(*i*) with deg(*i*) = number of adjacent edges of *i* and rec(*i*) = number of reciprocal edges of i (the *two* directions of one reciprocal edge are considered as *one* reciprocal edge).

The degree deg and the reciprocity rec are defined as:

$$\begin{array}{rcll} \deg\left(i\right) & =\underset{j\in N\backslash \left\{i\right\}}{\sum }a_{ij}+a_{ji} & & \text{(A43)}\\ \text{rec}\left(i\right) & =\underset{j\in N\backslash \left\{i\right\}}{\sum }a_{ij}\cdot a_{ji} & & \text{(A44)} \end{array}$$

For the directed and weighted case:

(A45) $$a_{ij}=\left\{\begin{array}{cc} 1\; & w_{ij}>0\\ 0\; & \text{else}\\ \; \end{array}\right.$$

#### A.2.23 Cluster coefficient (triangle based)

The triangle based cluster coefficient (Fagiolo, 2007) of a node *n* is the number of triangles around *n* divided by the maximum possible number. In this version of the cluster coefficient reciprocal edges to a neighbor of a node *n* can affect the cluster coefficient of node *n*. In the other version only edges between neighbors of *n* have an influence to the cluster coefficient of node *n*.

$$\begin{array}{rcll} C_{T}^{\to }=\frac{t^{\to }\left(i\right)}{t_{\max}\left(i\right)} & & & \text{(A46)}\\ C_{T}^{\vec{w}}=\frac{t^{\vec{w}}\left(i\right)}{t_{\max}\left(i\right)} & & & \text{(A47)} \end{array}$$

#### A.2.24 Cluster coefficient

Number of edges between the neighbors of a node divided by the maximum possible number. *C*^→^(*i*) refers to all neighbors of *i*.

(A48) $$C^{\to }\left(i\right)=\frac{1}{|N_{i}|\cdot \left(|N_{i}|-1\right)}\cdot \underset{\begin{array}{c} j,k\in N_{i}\\ j\neq k \end{array}}{\sum }a_{jk}$$

$C_{out}^{\to }\left(i\right)$ refers to the out-neighbors of *i*.

(A49) $$C_{out}^{\to }\left(i\right)=\frac{1}{|N_{i}^{out}|\cdot \left(|N_{i}^{out}|-1\right)}\cdot \underset{{}_{j\neq k}^{j,k\in N_{i}^{out}}}{\sum }a_{jk}$$

$C_{in}^{\to }(i)$ refers to the in-neighbors of *i*.

(A50) $$C_{in}^{\to }\left(i\right)=\frac{1}{|N_{i}^{in}|\cdot \left(|N_{i}^{in}|-1\right)}\cdot \underset{{}_{j\neq k}^{j,k\in N_{i}^{in}}}{\sum }a_{jk}$$

In the weighted case the *a_ij_* are replaced by the *w_ij_*.

#### A.2.25 Average cluster coefficient

(A51) $$C^{\to }=\frac{1}{n}\overset{n}{\underset{i=1}{\sum }}C_{i}^{\to }$$

and

(A52) $$C^{\vec{w}}=\frac{1}{n}\overset{n}{\underset{i=1}{\sum }}C_{i}^{\vec{w}}$$

#### A.2.26 Small-worldness *S*

(A53) $$S=\frac{\left(\frac{C}{C_{rand}}\right)}{\left(\bar{d}{\bar{d}}_{rand}\right)}$$

${\bar{d}}_{rand}$ and $C_{rand}$ are the parameters *C* and $\bar{d}$ of the Erdös Renyi randomizations.

#### A.2.27 Centrality

(A54) $$C_{D}=\frac{\overset{n}{\underset{i=1}{\sum }}deg_{\max}-deg\left(i\right)}{\left(n-1\right)\cdot \left(n-2\right)}=\frac{n\cdot deg_{\max}-2\cdot \ell }{\left(n-1\right)\cdot \left(n-2\right)}$$

This centrality (degree centrality) is defined for an undirected network based on undirected degrees. A directed or weighted version is not available yet. For the calculation the directed network is transferred to an undirected one.

#### A.2.28 Circle length (*LC*)

(A55) $$LC\left(i\right)=\left\{\begin{array}{cc} d\left(i,\;i\right),\; & d\left(i,\;i\right)<\infty \\ 0,\; & d\left(i,\;i\right)=\infty \\ \; \end{array}\right.$$

#### A.2.29 Eccentricity out

Eccentricity out, the output eccentricity of the vertex *i* is the maximum distance from *i* to any vertex.

(A56) $$Ecc^{out}\left(i\right)=\max\left\{d\left(i,j\right)\;|\;j\in N\wedge d\left(i,j\right)<\infty \right\}$$

#### A.2.30 Eccentricity in

Eccentricity in, the input eccentricity of the vertex *i* is the maximum distance from any vertex to *i*.

(A57) $$Ecc^{in}\left(i\right)=\max\left\{d\left(j,i\right)\;|\;j\in N\wedge d\left(i,j\right)<\infty \right\}$$

#### A.2.31 Cluster coefficient of second neighbors

The cluster coefficient of second neighbors (Hierarchical directed cluster coefficient of second (indirect) neighbors) *C*_2_(*i*) is the number of edges between the 2nd neighbors of node *i*, divided by the maximum possible number of edges. In the weighted case it is the sum of weights of the edges between the 2nd neighbors of node *i*, divided by the maximum possible sum. With

(A58) $$N_{2}\left(i\right)=\left(\underset{j\in N_{i}}{⋃}N_{j}\right)\backslash N_{i}^{+},$$

the set of second neighbors of node *i* is:

(A59) $$C_{2}\left(i\right)=\left\{\begin{array}{cc} \frac{1}{|N_{2}\left(i\right)|\cdot \left(|N_{2}\left(i\right)|-1\right)}\underset{\begin{array}{c} j,k\in N_{2}\left(i\right)\\ j\neq k \end{array}}{\sum }a_{jk}\; & \text{, if }|N_{2}\left(i\right)|>1\\ 0\; & \text{, otherwise} \end{array}\right.$$

In the weighted case the *a_ij_* are replaced by *w_ij_*.

#### A.2.32 Average neighbor degree

The non-weighted average neighbor degree *NB*(*i*) of node *i* is

(A60) $$\deg NB\left(i\right)=\frac{1}{\left|N_{i}\right|}\underset{j\in N_{i}}{\sum }\deg_{all}\left(\;j\right)$$

#### A.2.33 Weighted average neighbor degree

The weighted average neighbor degree *NB*(*i*) of node *i* is

(A61) $$\deg NB^{\vec{w}}\left(i\right)=\frac{1}{\left|N_{i}\right|}\underset{j\in N_{i}}{\sum }\deg_{all}^{w}\left(\;j\right)$$

#### A.2.34 Variation coefficient of neighbor degree

(A62) $$Loc\left(i\right)=\frac{\sqrt{\frac{1}{\left|N_{i}\right|}\underset{j\in N_{i}}{\sum }{\left(\deg_{all}\left(\;j\right)-\deg NB\left(i\right)\right)}^{2}}}{\deg NB\left(i\right)}$$

The weighted case is analog.

#### A.2.35 Locality index of node i [*Loc*(i)]

The locality index of node *i* is the fraction of edges adjacent to nodes in $N_{i}^{+}$ whose source and target lie in $N_{i}^{+}.$

(A63) $$Loc\left(i\right)=\frac{\underset{j\in N_{i}^{+}}{\sum }\;\underset{\begin{array}{c} k\in N_{i}^{+}\\ k\neq j \end{array}}{\sum }a_{jk}}{\underset{j\in N_{i}^{+}}{\sum }\;\underset{{}_{k\neq j}^{k\in N}}{\sum }a_{jk}}$$

The weighted case is analog. A value of 0 means that the node is isolated. The larger the value, the less edges connect the neighborhood of *i* to outside node. The maximum of one is reached if the neighborhood of *i* is not connected to outside nodes.

#### A.2.36 Closeness centrality out *CC^out^*(*i*)

The closeness centrality out with indices of nodes from which node i can be reached *RN^OUT^*(*i*) = {*j* ∈ *N*{*i*}|*d*(*i*, *j*) < ∞}

(A64) $$CC^{OUT}\left(i\right)=\frac{\left|RN^{OUT}\left(i\right)\right|}{\underset{j\in RN^{OUT}\left(i\right)}{\sum }\;d\left(i,j\right)}$$

#### A.2.37 Closeness centrality in *CC*^in^(*i*)

The closeness centrality in with indices of nodes which can be reached from node *i* *RN^IN^*(*i*) = {*j* ∈ *N*\{*i*}|*d*(*j*, *i*) < ∞}

(A65) $$CC^{IN}\left(i\right)=\frac{\left|RN^{IN}\left(i\right)\right|}{\underset{j\in RN^{IN}\left(i\right)}{\sum }\;d\left(\;j,i\right)}$$

#### A.2.38 Betweenness centrality (*BC*)

(A66) $$BC\left(i\right)=\frac{1}{\left(n-1\right)\left(n-2\right)}\cdot \underset{j,k\in N\backslash \left\{i\right\}}{\sum }\frac{\rho_{j,k}\left(i\right)}{\rho_{j,k}}$$

Where *ρ_j,k_* is the number of shortest paths from *j* to *k* and *ρ_j,k_*(*i*) is the number of shortest paths from *j* to *k* that pass through *i*. The directed and weighted definitions are the same.

#### A.2.39 Stress (*S*)

(A67) $$S\left(i\right)=\underset{j,k\in N\backslash \left\{i\right\}}{\sum }\rho_{j,k}\left(i\right)$$

The directed and weighted definitions are the same.

#### A.2.40 Central point distance (*CPD*)

(A68) $$CPD=\frac{1}{n-1}\overset{n}{\underset{i=1}{\sum }}\frac{BC_{max}-BC\left(i\right)}{BC_{max}}$$

Where $BC_{max}{=}_{i\in N}^{\max}\left\{BC\left(i\right)\right\}$ is the maximum betweenness centrality. The directed and weighted versions use the directed and weighted betweenness centralities.

#### A.2.41 Participation coefficient

The partition *M* = {*M*_1_, …, *M_m_*} is generated as described in the definition of modularity.

(A69) $$PC_{x}^{\to }\left(i\right)=1-\underset{M_{j}\in M}{\sum }{\left(\frac{\deg_{x}\left(i,M_{j}\right)}{\deg_{x}\left(i\right)}\right)}^{2}$$

with *x* ∈ {*in*, *out*, *all*} and

(A70) $$\deg_{in}\left(i,\;M_{j}\right)=\underset{k\in M_{j}\backslash \left\{i\right\}}{\sum }a_{ki}$$

(Number of edges from vertices of *M_j_* to *i*).

(A71) $$\deg_{out}\left(i,\;M_{j}\right)=\underset{k\in M_{j}\backslash \left\{i\right\}}{\sum }a_{ik}$$

(Number of edges from *i* to vertices of *M_j_*).

(A72) $$\deg_{all}\left(i,\;M_{j}\right)=\underset{k\in M_{j}\backslash \left\{i\right\}}{\sum }\left(a_{ik}+a_{ki}\right)$$

(Number of edges between *i* and vertices of *M${}_{j}$*).

(A73) $$PC_{x}^{\vec{w}}\left(i\right)=1-\underset{M_{j}\in M}{\sum }{\left(\frac{\deg_{x}^{w}\left(i,\;M_{j}\right)}{\deg_{x}^{w}\left(i\right)}\right)}^{2}$$

with the same *x* and weighted definitions of degrees. One has 0 ≤ *P*C(*i*) ≤ 1. If *PC*(*i*) = 1, the node *i* has no edges (in, out, all). If *PC*(*i*) = 0 all edges (in, out all) come from, go to, or stay in the same cluster. The larger *PC*(*i*) the more clusters are involved in the edges of node *i*.

#### A.2.42 Z-score/within module degree

Let *M_i_* be the module containing node *i*. deg*_x_*(*i*, *M_i_*) *x* ∈ {*in*, *out*, *all*} is explained in the definition of the participation coefficient.

(A74) $${\bar{\text{deg}}}_{x}\left(M_{i}\right)=\frac{1}{\left|M_{i}\right|}\underset{j\in M_{i}}{\sum }\deg_{x}\left(j,\;M_{i}\right)$$

is the mean and

(A75) $$\sigma_{\deg_{x}\left(M_{i}\right)}=\sqrt{\frac{1}{\left|M_{i}\right|}{\left(\underset{j\in M_{i}}{\sum }\deg_{x}\left(j,\;M_{i}-{\bar{\text{deg}}}_{x}\left(M_{i}\right)\right)\right)}^{2}}$$

the standard deviation of the within module *M_i_* degree distribution. Then the z-score is defined as

(A76) $$Z_{x}^{\to }\left(i\right)=\frac{\deg_{x}\left(i,\;M_{i}\right)-{\bar{\text{deg}}}_{x}\left(M_{i}\right)}{\sigma_{deg_{x}\left(M_{i}\right)}}$$

and analog

(A77) $$Z_{x}^{\vec{w}}\left(i\right)=\frac{\deg_{x}^{w}\left(i,\;M_{i}\right)-\bar{\deg_{x}^{w}}\left(M_{i}\right)}{\sigma_{deg_{x}^{w}\left(M_{i}\right)}}$$

with the weighted versions of the mean and standard deviation. A value above one or below minus one implies that a node has significantly more or less edges from, to, or from and to nodes in its cluster than the average node in its cluster has.

#### A.2.43 Eigenvector centrality

The eigenvector centrality *EC*(*i*) is the i-th component of the eigenvector with the largest corresponding eigenvalue of the adjacency matrix resp. weight matrix.

#### A.2.44 Shapley rating ϕ

The Shapley rating is a measure that provides information about the loss of connectivity following the removal of a node.

$$\begin{array}{rcll} SR\left(i\right) & =\underset{\hat{N}\subseteq N\backslash \left\{i\right\}}{\sum }\left(\left|SCC\left(\hat{N}\cup \left\{i\right\}\right)\right|-\left|SCC\left(\hat{N}\right)\right|\right) & & \\ & \;\cdot \frac{\left(n-|\hat{N}|-1\right)!\cdot |\hat{N}|!}{n!} & & \text{(A78)} \end{array}$$

Where $SCC\left(\hat{N}\right)$ is the set of strongly connected components of $\hat{N}.$ The smaller the value is, the more important is the node in the sense of connectivity of the graph. Because of the exponential number of subsets, this parameter can be approximated for large networks, only.

#### A.2.45 Radiality

The radiality of a node *Rad* is a measure of the distance of a node to all other nodes. Nodes that have a small radiality have larger distances to other nodes than those with a greater radiality.

#### A.2.46 Input radiality (*Rad_in_*)

The input radiality of a node *Rad_in_* is

(A79) $$Rad_{in}\left(i\right)=\frac{1}{n-1}\underset{\begin{array}{c} j\in N\\ d\left(j,i\right)<\infty \end{array}}{\sum }Diam+1-d\left(j,\;i\right)$$

In the weighted case the weighted distances are used.

#### A.2.47 Output radiality (*Rad_out_*)

The output radiality of a node *Rad_out_* is

(A80) $$Rad_{out}\left(i\right)=\frac{1}{n-1}\underset{\begin{array}{c} j\in N\\ d\left(i,j\right)<\infty \end{array}}{\sum }Diam+1-d\left(i,\;j\right)$$

In the weighted case the weighted distances are used.

#### A.2.48 Centroid value (*Cen*)

With *g_out_*(*i*, *j*) = |{*k* ∈ *N*|*d*(*i*, *k*) < *d*(*j*, *k*) < ∞}| and *g_in_*(*i*, *j*) = |{*k* ∈ *N*|*d*(*k*, *i*) < *d*(*k*, *j*) < ∞}| which are the number of nodes closer to node *i* than to node *j* with regard to In- and Out-distance, the centroid value is defined in the following.

#### A.2.49 Output centroid value (*Cen_out_*)

(A81) $$Cen_{out}\left(i\right)=\min\left\{g_{out}\left(i,\;j\right)-g_{out}\left(j,\;i\right)\;|\;j\in N\backslash \left\{i\right\}\right\}$$

#### A.2.50 Input centroid value (*Cen_in_*)

(A82) $$Cen_{in}\left(i\right)=\min\left\{g_{in}\left(i,\;j\right)-g_{in}\left(j,\;i\right)\;|\;j\in N\backslash \left\{i\right\}\right\}$$

In the weighted case the weighted distances are used. A value < 0 implies, that there exists a node that is closer to most other nodes. A value ≥ 0 implies, that this node is most central in the network. A value = 0 implies, that there are more than one most central nodes.

#### A.2.51 Page rank centrality (*PRC*)

*PRC*(*i*) = *r_i_* where *r* is the solution of the linear system

(A83) $$\left(I-\alpha \cdot A^{T}\cdot B\right)\cdot r=\frac{1}{n}\left(1-\alpha \right)\cdot \left(\;\begin{array}{c} 1\\ \vdots \\ 1 \end{array}\right)$$

with the damping factor α = 0.85, the identity matrix *I*, and the diagonal matrix *B*, whereby

(A84) $$b_{ii}=\left\{\begin{array}{cc} \frac{1}{\deg_{out}\left(i\right)},\; & deg_{out}\left(i\right)>0\\ 0,\; & \text{otherwise}\\ \; \end{array}\right.$$

In the weighted case the weight matrix *W* is used instead of *A* and the weighted version $deg_{out}^{\vec{w}}\left(i\right)$ of the outdegree.

#### A.2.52 Flow coefficient (*FC*)

Number of paths of length 2 between neighbors of a node *i* that pass node *i* divided by the maximum possible numbers of such paths.

(A85) $$FC\left(i\right)=\frac{1}{|N_{i}|\cdot \left(|N_{i}|-1\right)}\cdot \underset{\begin{array}{c} j,k\in N_{i}\\ j\neq k \end{array}}{\sum }a_{ji}\cdot a_{ik}$$

In the weighted case we define the flow coefficient as the sum of weights of paths of length 2 between neighbors of a node *i* that pass node *i* divided by the maximum possible sum.

(A86) $$FC^{\vec{w}}\left(i\right)=\frac{1}{2\cdot |N_{i}|\cdot \left(|N_{i}|-1\right)}\cdot \underset{\begin{array}{c} j\in N_{i}\\ w_{ji}>0 \end{array}}{\sum }\;\underset{{}_{w_{ik}>0}^{k\in N_{i}\backslash \left\{j\right\}}}{\sum }\left(w_{ji}+w_{ik}\right)$$

#### A.2.53 Average flow coefficient (*FC*)

Number of paths of length 2 between neighbors of a node *i* that pass node *i* divided by the maximum possible numbers of sub paths.

$$\begin{array}{rcll} FC & =\frac{1}{n}\overset{n}{\underset{i=1}{\sum }}FC\left(i\right) & & \text{(A87)}\\ FC^{\vec{w}} & =\frac{1}{n}\overset{n}{\underset{i=1}{\sum }}FC^{\vec{w}}\left(i\right) & & \text{(A88)} \end{array}$$

#### A.2.54 Subgraph centrality (*SC*)

$$\begin{array}{rcll} SC\left(i\right) & =\overset{\infty }{\underset{k=0}{\sum }}\frac{{\left(A^{k}\right)}_{ii}}{k!} & & \text{(A89)}\\ SC^{\vec{w}}\left(i\right) & =\overset{\infty }{\underset{k=0}{\sum }}\;\frac{{\left(W^{k}\right)}_{ii}}{k!} & & \text{(A90)} \end{array}$$

The subgraph centrality of the network is the average subgraph centrality of its nodes.

$$\begin{array}{rcll} SC & =\frac{1}{n}\overset{n}{\underset{i=1}{\sum }}\;SC\left(i\right) & & \text{(A91)}\\ SC^{\vec{w}} & =\frac{1}{n}\overset{n}{\underset{i=1}{\sum }}\;SC^{\vec{w}}\left(i\right) & & \text{(A92)} \end{array}$$

#### A.2.55 Undirected cyclic coefficient (*CyclC*)

The undirected cyclic coefficient as published by Kim and Kim (2005).

(A93) $$CyclC\left(i\right)=\frac{2}{|N_{i}|\cdot \left(|N_{i}|-1\right)}\cdot \underset{\begin{array}{c} \left(\;j,k\right)\in N_{i}\times N_{i}\\ j\neq k \end{array}}{\sum }\;\frac{1}{2+dist_{i}\left(\;j,k\right)}$$

With

(A94) $$dist_{i}\left(\;j,\;k\right)=\left\{\begin{array}{c} \text{length of the shortest path from }j\text{ to }k\;\\ \;\text{ that does not contains }i,\;\\ \text{if such a path exists}\;\\ \infty ,\;\text{otherwise}\;\\ \; \end{array}\right.$$

#### A.2.56 Directed cyclic coefficient (*CyclC^→^*)

A publication about the directed cyclic coefficient is unknown. The directed cyclic coefficient is implemented here as follows:

$$\begin{array}{rcll} CyclC^{\to }\left(i\right) & =\frac{1}{\left|N_{i}^{out}|\cdot |N_{i}^{in}|-|N_{i}^{out}\cap N_{i}^{in}\right|} & & \\ & \;\cdot \underset{\begin{array}{c} \left(\;j,k\right)\in N_{i}^{out}\times N_{i}^{in}\\ j\neq k \end{array}}{\sum }\;\frac{1}{2+dist_{i}\left(\;j,k\right)} & & \text{(95)} \end{array}$$

#### A.2.57 Directed weighted cyclic coefficient (${\text{CyclC}}^{{\;w}^{{}^{\;\;\;\;\;\;\to }}}$)

A publication about the directed weighted cyclic coefficient is unknown. The directed weighted cyclic coefficient is implemented here as follows:

$$\begin{array}{rcll} CyclC^{\vec{w}}\left(i\right) & =\frac{1}{\left|N_{i}^{out}|\cdot |N_{i}^{in}|-|N_{i}^{out}\cap N_{i}^{in}\right|} & & \\ & \;\cdot \underset{\begin{array}{c} \left(\;j,k\right)\in N_{i}^{out}\times N_{i}^{in}\\ j\neq k \end{array}}{\sum }\;\frac{1}{w_{ij}+w_{ki}+dist_{i}^{w}\left(\;j,k\right)} & & \text{(96)} \end{array}$$

with $dist_{i}^{w}\left(\;j,k\right)$ is the weighted version of *dist_i_*(*j*, *k*) with the weighted path length.

#### A.2.58 Cyclic network coefficient (*CyclC*^ →^)

The cyclic coefficient of the network is the average cyclic coefficient of its nodes:

(A97) $$CyclC^{\to }=\frac{1}{n}\overset{n}{\underset{i=1}{\sum }}\;CyclC^{\to }\left(i\right)$$

#### A.2.59 Independent cycle count (*CyC*)

The number of independent cycles is:

(A98) Cy=ϵ-n+1

The relative number of independent cycles is:

$$\begin{array}{rcll} CyC & =\frac{Cy}{Cy_{max}}=\frac{2Cy}{n\left(n+1\right)}=\frac{2\left(\epsilon -n+1\right)}{n\left(n+1\right)} & & \text{(A99)}\\ CyC^{\to } & =\frac{Cy}{n^{2}}=\frac{\epsilon -n+1}{n^{2}} & & \text{(A100)} \end{array}$$

#### A.2.60 Vulnerability (*V*)

The vulnerability *V* is the maximum relative decrease of the global efficiency removing a single node.

(A101) $$V=\underset{i\in N}{\max}\left\{\frac{GE-GE\left(i\right)}{GE}\right\}$$

Where *GE*(*i*) is the global efficiency of the graph (*N*\{*i*},{(*j*, *k*) ∈ *E* | *j* ≠ *i* ≠ *k*}) that originates by removal of node *i* and all edges adjacent to *i*. The weighted version is analog using the weighted global efficiencies.

#### A.2.61 Hubness

The hubness is the degree to which a node has output to authorities (Kleinberg, 1999).

#### A.2.62 Authoritativeness

The authoritativeness is the degree to which a node gets input from hubs (Kleinberg, 1999).

### A.3 Random models

The following random graph models are compared to the real network of the intrinsic amygdala connectivity. By comparing the average path length and the cluster coefficient of the models with the real network it is feasible to determine a model that is most similar to the real network.

#### A.3.1 Erdös Renyi graph

(A102) $$G\left(n,\;p\right)$$

where *n* is the number of vertices and *p* is the probability that an edge (*i*, *j*) exists, for all *i*, *j*. The degree distribution of the Erdös Renyi random graph is binominal in terms of

(A103) $$P\left(deg\left(v\right)=k\right)=\left(\;\begin{array}{c} n-1\\ k \end{array}\right)p^{k}{\left(1-p\right)}^{n-1-k}$$

#### A.3.2 Watts-Strogatz graph

The small-world model of Watts-Strogatz is a random graph generation model that provides graphs with small-world properties. The network (initially it has a non-random lattice structure) is build by linking each node to its <*k*> closest neighbors using are wiring probability *p*. Hence, an edge has the probability *p* that it will be rewired as a random edge. The number of rewired links can be estimated by:

(A104) pE=pN<k>∕2

#### A.3.3 Barabasi-Albert graph

The Barabasi-Albert graph is used to generate preferential attachments between nodes. The probability *p_i_* that the new node is connected to node *i* is

(A105) $$p_{i}=\frac{k_{i}}{\underset{j\in N}{\sum }k_{j}}$$

The degree distribution of a Barabasi-Albert network is scale-free following the power law distribution of the form:

(A106) $$P\left(k\right)\sim k^{-3}$$

#### A.3.4 Eipert graph

The modified Eipert model (EN: Eipert network) is based on the Barabasi-Albert graph. However, the algorithm starts at a fixed number of nodes and edges are added iteratively.

#### A.3.5 Ozik-Hunt-Ott graph

The Ozik-Hunt-Ott model (OHO; Ozik et al., 2004) is a small-world randomization approach that was modified for directed networks and a fixed number of edges. The OHO-model uses a growing mechanism in which all connections are made locally to topographical nearby regions.

#### A.3.6 Rewiring graph

The rewiring-models connects each target of an edge of a network to another target node.

#### A.3.7 Power law

(A107) $$P\left(k\right)=\alpha \cdot k^{-\gamma }$$

Δ is the deviation (error) of an empirical distribution of degrees from the power law function. A small Δ value means that the empirical distribution is similar with the power law function.

## Abbreviations

AA, AAA, anterior amygdaloid area; AAD, dorsal anterior amygdaloid area; AB, accessory basal nucleus; *AB*, accessory basal nucleus modified from de Olmos et al. (2004); ABm, accessory basal nucleus magnocellular division; ABmc, accessory basal nucleus magnocellular division; ABp, accessory basal nucleus parvicellular division; ABpc, accessory basal nucleus parvicellular division; ACo, layers 1-2 = anterior cortical nucleus; ACo, anterior cortical amygdaloid nucleus; AHA, amygdalohippocampal area; AHAl, amygdalohippocampal area lateral division; AHAm, amygdalohippocampal area medial division; AHi, amygdalohippocampal area; AHiAL, amygdalohippocampal area anterolateral; AHilp, Amygdalohippocampal area lateral part; AHilp, amygdalohippocampal area lateral part; AHimp, amygdalohippocampal area medial part; AHimp, amygdalohippocampal area medial part; AHiPM, amygdalohippocampal area, posteromedial; APir PM, amygdalopiriform transition area posteromedial part; AStrrc, amygdalostriatal transition area rostrocaudal part; B, basal nucleus; BA, bed nucleus accessory olfactory tract; BAOT, bed nucleus of the accessory olfactory tract; BAOT*, bed nucleus of accessory olfactory tract; Bi, basal nucleus intermediate division; BL, BLA, basolateral nucleus; BLA*, anterior basolateral nucleus; BLAa, basolateral nucleus, anterior part; BLAp, basolateral nucleus, posterior part; BLPLP, posterior basolateral nucleus lateral part; BLV, ventral basolateral nucleus; BM, BMA, basomedial nucleus; BMA, layer 3 = Anterior basomedial nucleus; BMA, anterior basomedial nucleus; BMAa, layer 3 = Basomedial nucleus, anterior part; BMAp, basomedial nucleus, posterior part; Bmc, basal nucleus magnocellular division; Bmc, basal nucleus magnocellular part; BMP, posterior basomedial nucleus; BMP, posterior basomedial nucleus; *Bnc*, basal nucleus modified from de Olmos et al. (2004); Bnci, basal nucleus intermediate division; Bpc, BPc, basal nucleus parvicellular division; BSTAD, bed nucleus of the stria terminalis anterior dorsal area; BSTAV, bed nucleus of the stria terminalis anterior ventral area; BSTdm, bed nucleus of the stria terminalis anterior division dorsomedial nucleus; BSTif, bed nucleus of the stria terminalis interfascicular nucleus; BSTLD, bed nucleus of the stria terminalis lateral division dorsal part; BSTLP, bed nucleus of the stria terminalis lateral division posterior part; BSTLV, bed nucleus of the stria terminalis lateral division ventral part; BSTmc, bed nucleus of the stria terminalis magnocellular nucleus; BSTMPL, bed nucleus of the stria terminalis medial division posterolateral part; BSTpr, bed nucleus of the stria terminalis principal nucleus; BSTSl, supracapsular bed nucleus of the stria terminalis lateral part; BSTt, bed nucleus of the stria terminalis transverse nucleus; CE, Ce, CEA, central nucleus; CE*_l_*, central nucleus lateral division; CEA*_l_*, central nucleus, lateral part; CEAc, central nucleus, capsular part; CEAm, central nucleus, medial part; Cec, CEc, central nucleus capsular division; CeCD, central amygdaloid nucleus caudal division; CeI, central amygdaloid nucleus intermediate division; CeI, CEi, central nucleus intermediate division; CeL, central nucleus, lateral division; CeLC, central nucleus, lateral division, capsular; CeLCn, central nucleus, lateral division, central; CEm, central nucleus medial division; CeM, central nucleus, medial division; Co, cortical nucleus; COA, cortical nucleus of the amygala; COa, anterior cortical nucleus; COAa, layers 1-2 = Cortical nucleus, anterior part; COAp, posterior amygdaloid nucleus; COApl, cortical nucleus, posterior part, lateral zone; COApm, cortical nucleus, posterior part, medial zone; COApm, posterior amygdaloid nucleus medial part; COp, posterior cortical nucleus; EN, Eipert network; Fu, bed nucleus of the stria terminalis fusiform part; I, intercalated nuclei, Intercalated nuclei of the amygdala; IA, intercalated nuclei; IM, intercalated masses; i.n., included in; IPACL, interstitial nucleus of the posterior limb of the anterior commissure lateral part; IPACM, interstitial nucleus of the posterior limb of the anterior commissure medial part; I*, intercalated masses; L, La, lateral nucleus; LAM, lateral nucleus medial part; LaVL, lateral nucleus ventrolateral nucleus; LaVM, ventromedial lateral nucleus; LaVM, ventromedial part of the lateral nucleus; Ldl, LaDL, lateral nucleus dorsolateral division; Level, level of subdivision (first level is the supranuclear division of the amygdala); Lm, lateral nucleus medial division; LOT, nucleus of the lateral olfactory tract; LOTL2, nucleus of the lateral olfactory tract layer 2; LOTL3, nucleus of the lateral olfactory tract layer 3; Lvl, lateral nucleus ventrolateral division; M, medial nucleus; Mc, medial nucleus caudal division; Mcd, dorsal part of central division of the medial nucleus; Mcv, ventral part of central division of the medial nucleus; Me, MEA, medial nucleus; MEAad, medial nucleus, anterodorsal part; MEAav, medial nucleus, anteroventral part; MeAD, anterodorsal medial nucleus; MeAD, medial amygdaloid nucleus anterodorsal part; MEApd, medial nucleus, posterodorsal part; MEApv, medial nucleus, posteroventral part; MeAV, anteroventral medial nucleus; MeC, medial amygdaloid nucleus caudal part; MeCd, medial nucleus central division; MeCD, medial amygdaloid nucleus central dorsal part; MeCV, medial amygdaloid nucleus central ventral part; MePD, medial amygdaloid nucleus posterodorsal part; MePD, posterodorsal medial nucleus; MePV, medial amygdaloid nucleus posteroventral part; MePV, posteroventral medial nucleus; MeRo, medial amygdaloid nucleus rostral part; Mr, medial nucleus rostral division; NLOT, nucleus of the lateral olfactory tract; PA, posterior nucleus amygdala; PAC, periamygdaloid cortex; PACm, periamygdaloid complex medial division; PACm, periamygdaloid cortex, medial division; PACs, periamygdaloid complex sulcal division; PACs, periamygdaloid cortex, sulcal division; PLCo, posterolateral cortical nucleus; PMCo, posteromedial cortical nucleus; SLEAc, central division of sublenticular extended amygdala; SV, Subventricular nucleus; w.n.s., with no subdivisions.
